# Development and evaluation of two-parameter linear free energy models for the prediction of human skin permeability coefficient of neutral organic chemicals

**DOI:** 10.1186/s13321-021-00503-5

**Published:** 2021-03-19

**Authors:** Sana Naseem, Yasuyuki Zushi, Deedar Nabi

**Affiliations:** 1 Institute of Environmental Sciences and Engineering (IESE), National University of Sciences and Technology (NUST), H-12, Islamabad, Pakistan; 2 Research Institute of Science for Safety and Sustainability, National Institute of Advanced Industrial Science and Technology (AIST), 16-1 Onogawa, Tsukuba, Ibaraki 305-8569 Japan; 3 College of Health Sciences, Jumeira University, Dubai, United Arab Emirates

**Keywords:** Skin permeability, Linear Free Energy Relationship (LFER) Modeling, Abraham solvation model, GC × GC model, Complex mixtures, Dermal Permeability Coefficient Program (DERMWIN™), QSARs

## Abstract

The experimental values of skin permeability coefficients, required for dermal exposure assessment, are not readily available for many chemicals. The existing estimation approaches are either less accurate or require many parameters that are not readily available. Furthermore, current estimation methods are not easy to apply to complex environmental mixtures. We present two models to estimate the skin permeability coefficients of neutral organic chemicals. The first model, referred to here as the 2-parameter partitioning model (PPM), exploits a linear free energy relationship (LFER) of skin permeability coefficient with a linear combination of partition coefficients for octanol–water and air–water systems. The second model is based on the retention time information of nonpolar analytes on comprehensive two-dimensional gas chromatography (GC × GC). The PPM successfully explained variability in the skin permeability data (*n* = 175) with *R*^2^ = 0.82 and root mean square error (*RMSE*) = 0.47 *log* unit. In comparison, the US-EPA’s model DERMWIN™ exhibited an *RMSE* of 0.78 *log* unit. The Zhang model—a 5-parameter LFER equation based on experimental Abraham solute descriptors (ASDs)—performed slightly better with an *RMSE* value of 0.44 *log* unit. However, the Zhang model is limited by the scarcity of experimental ASDs. The GC × GC model successfully explained the variance in skin permeability data of nonpolar chemicals (*n* = 79) with *R*^2^ = 0.90 and *RMSE* = 0.23 *log* unit. The PPM can easily be implemented in US-EPA’s Estimation Program Interface Suite (EPI Suite™). The GC × GC model can be applied to the complex mixtures of nonpolar chemicals.

**Supplementary Information:**

The online version contains supplementary material available at 10.1186/s13321-021-00503-5.

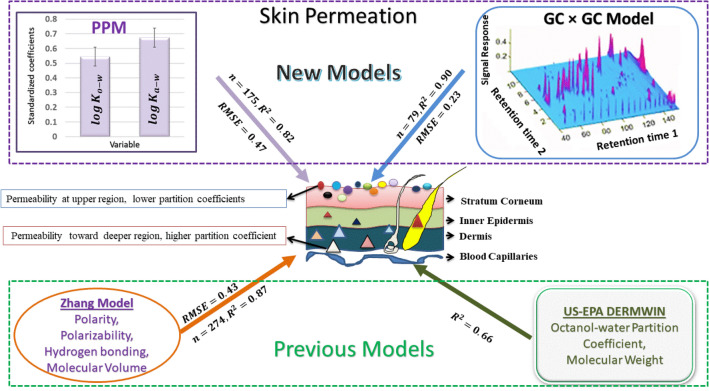

## Introduction

The skin, being the largest organ, is prone to exposure of organic chemicals found in environmental media [1, 2], and occupational settings [3], and in consumer products [4, 5]. The permeability coefficient ($K_{p}$) is a key parameter for the assessment of dermal exposure to these chemicals. Currently, the experimental data of $K_{p}$, available in the public domain, are limited to only a few hundred organic chemicals [6]. Experimental methods based on various in vivo and in vitro techniques [7] are expensive, laborious, and have ethical implications of animal-testing. Therefore, there is a growing interest in developing fast and easy estimation methods for skin permeability.

Estimation methods, based on quantitative structure–activity relationships (QSARs), exploit the relationships between the permeability coefficient, and the descriptors of lipophilicity and diffusivity [7]. Several QSARs were developed using octanol–water partition coefficient ($K_{ow}$) and molecular weight ($MW$) as the respective descriptors of lipophilicity and diffusivity [8]. The dermal permeability modeling program (DERMWIN™), developed by the United States Environmental Protection Agency (US-EPA), is built on one of such relations. The DERMWIN™ uses Eq. 1 for the estimation of skin permeability coefficient (cm/h) for a diverse set of chemicals. This module is freely available in the Estimation Programs Interface (EPI) Suite ™ Version 4.11 [9]**.**

1 $${ }log K_{p } = - 2.80 + 0.66log K_{ow} - 0.0056 MW$$

The documentation of DERMWIN™ describes $R^{2} = 0.66$ for this model implying that the two parameters, $log K_{o - w}$ and molecular weight ($MW$), are not enough to account for remaining 34% variability in the skin permeation data. The On-line DERMWIN™ User’s Guide does not provide more information on types of datasets and regression statistics for this model. The model based on $log K_{ow}$ and $MW$ can yield errors up to one to two orders of magnitude compared to experimental data [10]. The inadequacy of DERMWIN™ may be attributed to the fact that the octanol is not an exact surrogate phase for the dermal lipid, and it does not reflect all types of interactions that chemicals experience with structural proteins present in the stratum corneum layer of skin. This requires the improvement of the model by including a descriptor that would take care of the interactions not accounted for by the octanol phase.

Zhang and coworkers developed a poly-parameter Linear Free Energy Relationship (LFER) model (Eq. 2) based on Abraham solute descriptors to estimate skin—water permeability coefficients [11]. The Zhang model shows that intermolecular interaction parameters such as solute size, polarity/polarizability, hydrogen-bond interactions and ionizability of chemical play a significant role in the estimation of $K_{p}$.

2 $$\begin{aligned} log { }K_{p} & = - 5.328\left( { \pm 0.071} \right) + { }0.137( \pm 0.082)E - 0.604\left( { \pm 0.057} \right)S \\ & \quad - 0.338\left( { \pm 0.094} \right)A - 2.428\left( { \pm 0.090} \right){\text{B}} + 1.797\left( { \pm 0.079} \right){\text{V }} \\ & \quad - 1.485\left( { \pm 0.121} \right)J^{ + } + 2.471\left( { \pm 0.113} \right)J^{ - } \\ \end{aligned}$$

$$n = 274,{ }R^{2} = 0.866,{ }Q^{2} = 0.858,{ }RMSE = 0.432{ }$$

In Eq. 2, $E$ describes the polarizability of molecule, $S$ shows the mix of polarity/polarizability interaction of the solute, $A$ describes the hydrogen bond donating capacity, $B$ denotes the hydrogen bond accepting capacity, $V$ expresses the volume of a solute in McGowan characterization (cm^3^/mol)/10, and $J^{ + }$, $J^{ - }$ are descriptors which are specific for anions and cations respectively [11–20]. For neutral molecules, the values of $J^{ + }$, $J^{ - }$ descriptors are equal to zero. Hence, Eq. 2 becomes five parameter LFER for neutral molecules. In Eq. 2, $K_{p}$ is given in unit of cm/s. The explanatory power of Eq. 2 is higher than that of DERMWIN™ but at the cost of expensive experimental input parameters. Experimental data of Abraham solute descriptors (ASDs) comprises of < 8000 chemicals [21]. This calls for a model that can accurately estimate $K_{p }$ for the chemicals for which the ASDs are not available.

Previous studies demonstrated the potential of chromatographic techniques such as liquid chromatography [22] and micellar chromatography [23] for the estimation of skin permeation. However, these techniques are not easy to apply on the complex mixtures. Comprehensive two-dimensional gas chromatography (GC × GC) is a powerful technique that is capable of separating hundreds of thousands of chemicals in complex mixtures [24]. Scientists were able to identify known skin penetrants in environmental samples such as the household dust using comprehensive two-dimensional liquid chromatography coupled with time-of-flight mass spectrometry [25]. In addition to its separation power, recent studies [26–28] demonstrated the potential of GC × GC in chemical risk assessment. Several environmental partitioning and diffusion-related properties ($log K$) of nonpolar complex organic mixtures were successfully estimated using LFER based on two solute parameters ($u_{1,i}$ and $u_{2,i}$), which were derived from the first- and second-dimension retention times of analytes on GC × GC chromatogram. The GC × GC model (Eq. 3) was first theoretically calibrated for 32 properties using a set of 79 nonpolar model chemicals, and then validated experimentally with a set of 52 nonpolar chemicals analyzed on the GC × GC instrument.

3 $$log K = \lambda_{1} u_{1,i} + \lambda_{2} u_{2,i} + \lambda_{3}$$

where $\lambda_{1}$, $\lambda_{2} , \lambda_{3}$ are specific to partitioning system. The power of GC × GC model is that the estimates of properties can be applied directly on to the detected nonpolar chemicals in environmental mixtures.

The skin permeability coefficient of a chemical through stratum corneum is related to the partition coefficient and diffusivity via Eq. 4.

4 $${ }K_{p } = \frac{{K_{m} D}}{h}$$

where $K_{m}$ is the partition coefficient between the stratum corneum and the vehicle, $D$ is the effective diffusion coefficient of the chemical through the stratum corneum, and $h$ is the apparent thickness of the stratum corneum. Previously, Eq. 3 was quite successful in predicting the aqueous diffusivity, and the partitioning of nonpolar chemicals from lipid and protein (important phases of stratum corneum) to water. Therefore, we expect that Eq. 3 can explain the variability in skin permeability of nonpolar chemicals.

In this study, we hypothesized following: (1) a linear combination of $K_{ow}$ and $K_{aw}$ (air–water partition coefficient) better explains the variability of skin permeation data as compared to DERMWIN™ equation because $K_{aw}$ brings in significant information about hydrogen-bonding interaction [29], which is not sufficiently provided by the combination of $K_{ow}$ and $MW$. (2) Given success of the GC × GC model with rate-related properties in previous studies, the GC × GC instrument provides suitable solute descriptors to model skin permeability of nonpolar complex mixtures.

## Materials and methods

### Data source and analysis

The experimental values of skin permeability coefficient ($K_{p}$) comprising 247 chemicals were taken from compilation given in the previous work [11]. We excluded ionized species from this data because our proposed models, PPM and GC × GC model, can theoretically account for the intermolecular interactions for neutral organic chemicals only. This resulted into a data size of 175 neutral organic chemicals, which are shown in Additional file 1: Table S1 along with the values of ASDs.

For calibration and evaluation of the PPM, the experimental $K_{ow}$ and $K_{aw}$ values were available only for 68 chemicals in $K_{p}$ dataset. Therefore, we calibrated the PPM with the *log* $K_{ow}$ and *log* $K_{aw}$ values estimated using Abraham solvation model (ASM) equations [30, 31]. Compared to other estimation approaches, the ASM equations are known to provide accurate estimates of $log K_{ow}$ and $log K_{aw}$ [30, 31]. To further evaluate the accuracies, we compared the predictions of ASM [32] and EPI-Suite with the experimental data of $log K_{ow}$ and $log K_{aw}$ reported in reference [32] (data not shown). When compared to same experimental data ($n = 314$), the ASM [32] provides more accurate estimates $log K_{ow}$ ($RMSE = 0.15$) compared to KOWWIN v1.69 ($RMSE = 0.24$). The ASM equation for $log K_{aw}$ [32] performed much better ($RMSE = 0.12$) compared to Henrywin v3.21 ($RMSE = 0.40$ log unit), when the predictions of these models were compared with the same set of experimental data ($n = 390$). Hence, the experimental values, when available, should be preferred over ASM predicted values, which in turn should be preferred over the EPI-Suite predicted values of $log K_{ow}$ and $log K_{aw}$.

The PPM was also evaluated with the input of experimental and EPI Suite™ (KOWWIN ver 1.68 and HenryWin ver 3.2) [9] estimated $K_{ow}$ and $K_{aw}$ values. The experimental and estimated values of $K_{ow}$ and $K_{aw}$ for the final datasets are shown in Additional file 1: Table S2. Once developed and evaluated rigorously using ASM predicted values $log K_{ow}$ and $log K_{aw}$, PPM does not require the ASDs any longer. The $log K_{p}$ values for chemicals—for which the ASDs are not available—can be calculated with the input of $log K_{ow}$ and $log K_{aw}$ values in the PPM, which can be either measured in laboratory, or can be found in existing published experimental databases or can be predicted reliably using estimation approaches. Generally, the measurement of $log K_{ow}$ and $log K_{aw}$ values is relatively easier than the measurement of ASDs in laboratory.

Lastly, we fitted the PPM to a dataset comprising only the experimental values of $log K_{p}$, $log K_{ow}$ and $log K_{aw}$ ($n = 68$). We also tested the fitting of PPM on the dataset ($n = 175$) comprising experimental $log K_{p}$ values and EPI Suite™ predicted *log* $K_{ow}$ and *log* $K_{aw}$ values. The fitting coefficients and regression statistics of the PPM obtained after such trainings were compared to those of the PPM trained on the dataset ($n = 175$) comprising experimental $log K_{p}$ values and ASM predicted *log* $K_{ow}$ and *log* $K_{aw}$ values.

Besides $K_{ow}$ and $K_{aw}$, we included other descriptors such as molecular weight ($MW$), organic carbon to water partition coefficient ($K_{ocw}$), bioconcentration factor ($BCF$), diffusion constant for water ($D_{w}$) and for ethanol ($D_{ethanol}$) to inspect their explanatory power for the $K_{p}$ data. The data for these additional descriptors were taken from different published sources [9, 33–35].

The experimental dataset of $K_{p}$, used to develop the PPM model, was diverse and spanned 7 orders of magnitude (Additional file 1: Table S1). The dataset contains chemicals with diverse structures and comprises of chemical families such as steroids, alcohol, acids, amines, amides, carbonyls, esters, urea, carboxylic acids, ether, halides, nitriles, nitro compounds and nonpolar organic compounds. The partition coefficients, $K_{ow}$ and $K_{aw}$, used for the calibration of the PPM, traversed diversified ranges.

For calibration and evaluation of the GC × GC Model, the calibration dataset was taken from previous study [26] because it was formulated in a way that represented nonpolar intermolecular interactions in a balanced way. This calibration dataset comprised of 79 chemicals (Additional file 1: Table S3), which spanned several nonpolar chemical families. The representativeness of the calibration dataset was further corroborated by the singular value decomposition (SVD) analysis, which was performed on six ASDs of 79 chemicals present in the calibration dataset. The SVD analysis indicated the first dimensions account for more than 99% of variance [26].

Next, the two new solute parameters, $u_{1}$ and $u_{2}$, of 79 chemicals in the calibration set were obtained by transforming the gas-stationary phase partition coefficient for the first and second dimension of the GC × GC. The values of the gas-stationary phase partition coefficient for these 79 chemicals were estimated using Abraham solvation model equations published for the relevant stationary phases [36]. The GC × GC based two-parameter LFER (Eq. 3) for skin permeation was developed with the $u_{1}$ and $u_{2}$ as independent variables and $logK_{p}$ as a dependent variable using multiple linear regression.

Finally, the above fitted GC × GC model was validated independently using a previously published [26] values of $u_{1}$ and $u_{2}$ for a set of 52 nonpolar chemicals (Additional file 1: Table S4). The solute parameters, $u_{1}$ and $u_{2}$ for this set were obtained by transforming first- and second-dimension retention times of nonpolar analytes measured on the GC × GC instrument [26, 27]. This validation set differed from the training set in the sense that $u_{1}$ and $u_{2}$ values of calibration set were obtained theoretically, while those of validation sets were obtained experimentally by analyzing these chemicals on the GC × GC instrument.

The experimental values of $K_{p}$ for the nonpolar chemicals in the training and validation sets for the GC × GC model were not available. Even though Zhang model is limited by scarce experimental data and non-applicability on the complex mixture, Zhang model provides—within these limitations—the most accurate predictions ($RMSE = 0.432$) compared to other existing LFERs. Due to lack of experimental data, we resorted to using the predicted values of $logK_{p}$ for developing the GC × GC model (Additional file 1: Tables S3 and S4). Once trained and validated robustly, the GC × GC model does not require the experimental ASDs anymore. Contrary to Zhang Model, the GC × GC model can now be applied on nonpolar complex mixtures. For the GC × GC model, users only need $u_{1}$ and $u_{2}$ for nonpolar chemical of interest, which can easily be determined by analyzing the nonpolar chemicals on the GC × GC instrument. The approach used to develop the GC × GC model is further elaborated in Additional file 1: Figure S1.

The training and validation datasets for the GC × GC model comprise of nonpolar chemicals only, which includes representatives of chemical families such as *n*-alkanes, cycloalkanes, cycloalkenes, halogenated alkanes, halogenated alkenes, benzene, linear alkylbenzenes, halogenated benzenes, polycyclic aromatic hydrocarbons (PAHs), polychlorinated biphenyls (PCBs), polybrominated diphenyl ethers (PBDEs), and polychlorinated naphthalenes (PCNs), and organochlorine pesticides.

### Statistical analysis

The statistical analyses such as multiple linear regression, cross validation tests, principle component analysis (PCA) were carried out using R-computational environment (3.5.3) [37] and XLSTAT (2018) [38]. The selection of significant and optimum number of descriptors was done using stepwise multiple linear regression based on the statistical criteria such as Student's t-test, Akaike information criteria, variance inflation factor. To delineate the domain of applicability, and to identify the influential values in the training datasets, the regression diagnostics such as studentized residuals, hat values and Cook’s distance were applied to each model (Additional file 1: Tables S5, S6, Figures S2, S3). Standard errors of beta-coefficients in all models were estimated using the bootstrapping technique (Additional file 1: Tables S7, S8). Cross-validation tests such as K-nearest neighbors, K-fold (*n* = 10), repeated K-fold (*n* = 10, *repeat* = 3), leave-one-out and bootstrapping (*n* = 1000) were performed for each model to evaluate the robustness (Additional file 1: Section S1, Table S9, S10). The PCA test was used to identify the contribution of all variables in the principal components.

## Results and discussion

### Justification of 2P-LFER

**Fig. 1:**
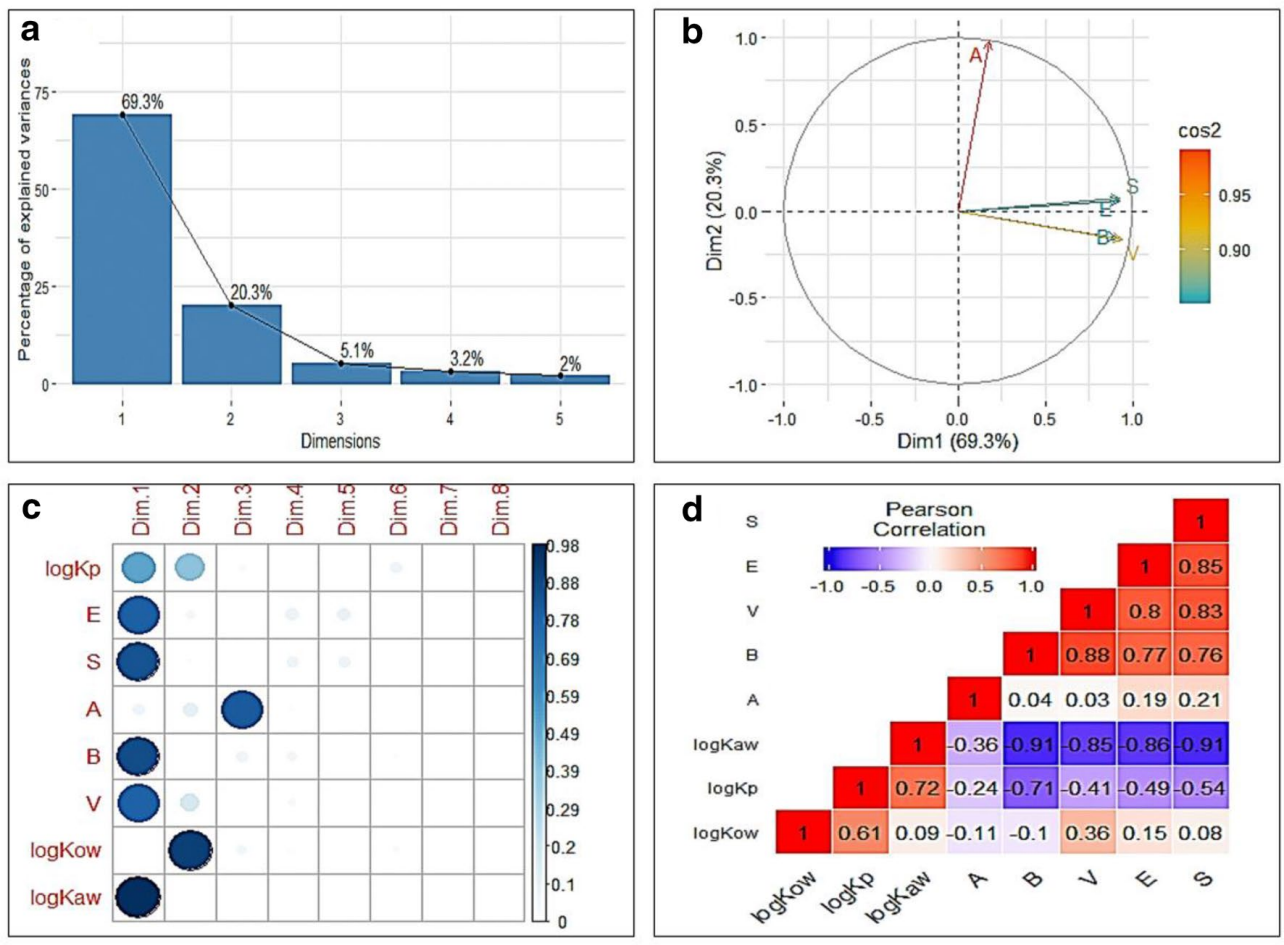
Dimensionality analysis for the PPM training set. Top panels show the results obtained by the Principal Component Analysis (PCA) ran on 175 × 5 matrix, [*E S A B V*], of Abraham solute descriptors for the training set of the Zhang Model in the form of **a** Scree Plot of eigenvalues (i.e., the amount of variation retained by each principal component), and **b** the correlation circle showing the relationship and quality of representation, square cosine (cos2), of variables in first two dimensions. Lower panels show **c** the distribution of quality of representation, Cos2, into 8 dimensions and **d** the correlogram of the correlation matrix, obtained respectively by the PCA and Pearson correlation analysis of 175 × 8 matrix, [*E S A B V log* $K_{ow}$ *log* $K_{aw}$ $log K_{p}$]. In **b**, the length of arrowed line from the origin shows the quality of representation of variable. Angles between the arrowed lines show the degree of correlations: Descriptor *A* is almost orthogonal to *E*, *S*, *B* and *V* descriptors, which are mutually positively correlated. In **c**, color intensity and size of the circle are proportional to the quality of presentation of a variable. In **d**, blue and red color respectively show positive and negative correlations between the pair. The value of correlation coefficient for each pair of variables is shown in each square. All correlations, shown here, were statistically significant (*p* < 0.05). In **b**, **c**, Dim. stands for the dimension

As a starting point for developing a parsimonious LFER model, we propose that skin permeation of neutral organic chemicals may be adequately estimated by the use of only two parameters, $K_{ow}$ and $K_{aw}$. To explore this hypothesis, we analyzed the information content contained in Abraham solute descriptors (ASDs) of the training set used to develop the Zhang Model. For neutral organic chemicals, the Zhang model shows that five dimensions of information are needed to successfully explain the variability in the skin permeability data. However, the PCA on 175 × 5 matrix, [*E S A B V*], of ASDs of the training set of the Zhang Model shows that the first two of total five dimensions encode 89.65% of information (Fig. 1a). The first dimension (principal component) is found to be formed by the linear combination of *E*, *S*, *B*, and *V*, with negligible contributions from *A* descriptor. The second dimension is represented mainly by *A* descriptor with very minor contribution from other ASDs (Fig. 1b). This indicates the possibility for the development of a parsimonious model based on two parameters without much loss of information.

Dimensionality analysis of the Zhang model set led us to the next important question of the study: what could be the two appropriate descriptors that would correspond to the first two dimensions of the PCA? The search for the appropriate descriptors started with the following considerations: these descriptors (i) should be easily accessible, (ii) have either large experimental database available or can easily be measured in laboratory or can be estimated using computationally-inexpensive but accurate methods, (iii) should sufficiently account for the changes in free energy due to transfer of molecule from water to skin. As shown below, the partition coefficients for octanol–water and air–water systems qualified for these considerations. To find the answer, we inspected the information loading resulting from the PCA of 175 × 8 matrix, [*E S A B V log* $K_{ow}$ *log* $K_{aw}$ $log K_{p}$], in the principal components. The first two of these total 8 dimensions correspond to 81.13% variability of the dataset (data not shown). Skin permeability coefficient was partitioned almost entirely between the first two dimensions. The partition coefficients for octanol–water and air–water (*log* $K_{ow}$ and *log* $K_{aw}$) were also apportioned almost entirely in the first two dimensions, respectively (Fig. 1c).

The correlation plot of all variables ($K_{p}$, ASDs, $K_{ow}$ and $K_{aw}$) indicates that $K_{ow}$ and $K_{aw}$ captures the important intermolecular interactions, otherwise coded in the ASDs, to describe the $K_{p}$ (Fig. 1d). Further, $log K_{ow}$ and $log K_{aw}$ are almost mutually orthogonal (Pearson correlation coefficient, $r = 0.09$), implying that both descriptors would deliver independent information to build a robust model for the skin permeability. Both descriptors, $log K_{ow}$ and $log K_{aw}$, shows strong correlations ($r = 0.61$ and $r = 0.72$, respectively) with $log K_{p}$. Practically, the suitability of $K_{ow}$ and $K_{aw}$ is desirable because these properties have a wider experimental database and quicker estimation approaches than those available for the ASDs [9, 30, 31]. Taken together, above results indicate that $K_{ow}$ and $K_{aw}$ are appropriate alternative parameters to describe the permeability variability for neutral organic molecules.

#### Two-parameter partitioning model

**Fig. 2:**
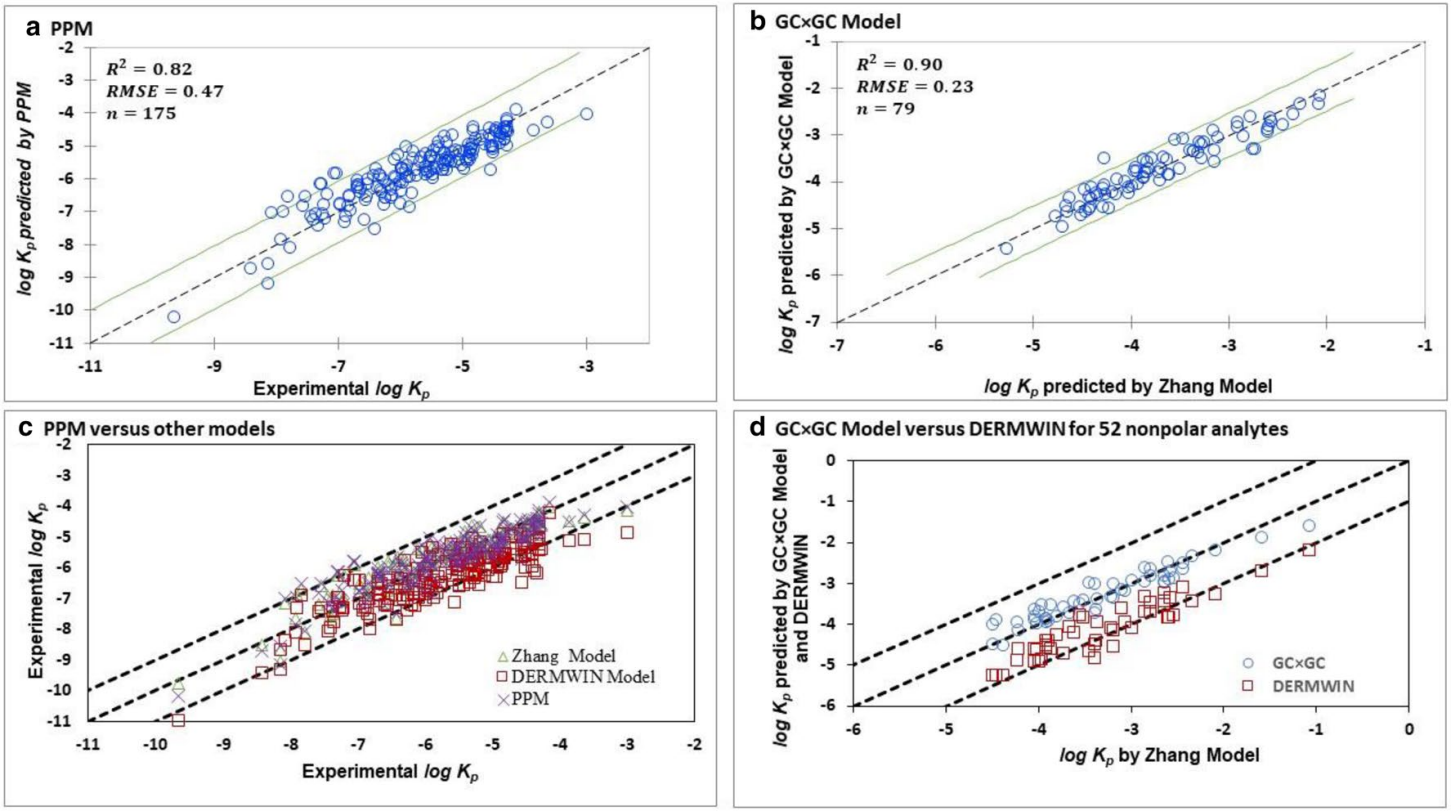
Linear regression plot for **a** Two-Parameter Partitioning Model (PPM), and **b** GC × GC Model. Upper and lower green lines bound 95% confidence interval around the regression line (dotted black line in the middle). Lower panels show (**c**) scatterplot obtained by comparing the prediction of $log K_{p}$ from three models, Zhang model (green triangles), DERMWIN™ (red square) and PPM (purple crosses), with the experimental values ($n = 175$). **d** The result of independent validation of the GC × GC Model obtained by comparing the predictions (green circles) for 52 nonpolar chemicals—which were analyzed on the GC × GC—with the predictions of the Zhang model. Predictions of DERMWIN™ (red squares) also shown for comparative purpose. In the lower panels, the dotted line in the middle shows 1:1 agreement, and upper and lower dotted lines indicate 1:2 agreement between the reference and predicted values

The PPM, based on a relationship of $log K_{p}$ with a linear combination of *log* $K_{ow}$ and *log* $K_{aw}$, successfully described 82% of variation in the $log K_{p}$ data (Eq. 5 and Fig. 2a).

5 $$log K_{p} = - 5.41 \left( { \pm 0.08} \right) + 0.46\left( { \pm 0.03} \right)log K_{ow } + 0.14 \left( { \pm 0.007} \right)log K_{aw }$$

$$n = 175,{ }R^{2} = 0.82,{\text{ Adj}}.{ }R^{2} = 0.82,{ }Q^{2} = 0.81,{ }RMSE = 0.47,PRESS{ }RMSE = 0.48$$

Here, the values of $K_{ow}$ and $K_{aw}$, used to train Eq. 5, were estimated by the respective ASM equations [30, 31]. Where, $n,{ }R^{2} ,{\text{ Adj}}.{ }R^{2} ,{ }Q^{2} ,{ }RMSE$ and $PRESS{ }RMSE$ respectively denote number of experimental values of $log K_{p}$, coefficient of determination, adjusted coefficient of determination, leave-one-out cross-validated $R^{2}$, root mean squared error and predicted residual error sum of squares, respectively.

Results of four independent cross-validation tests indicate that model (Eq. 5) is internally valid for predictive purpose (Additional file 1: Table S9). With the input of the limited experimental data of $K_{ow}$ and $K_{aw}$ values (*n* = 68), Eq. 5 exhibited good agreement between the experimental and predicted values of $log K_{p}$ ($RMSE = 0.36$ *log* units). Finally, we tested the performance of the PPM by inputting $K_{ow}$ and $K_{aw}$ values (*n* = 175) that were estimated respectively from the KOWWIN 1.68 and HenryWin 3.2 modules of EPI Suite™ 4.1. This yielded an $RMSE = 0.60$ *log* unit, which is better than the one ($RMSE = 0.82$) observed for the DERMWIN™ when compared with the same experimental data (*n* = 175). These statistics suggest that the PPM can be integrated in the EPI Suite™ as a better alternative to DERMWIN™ (Fig. 2c).

For external validation, the PPM full dataset (*n* = 175) was split randomly into a training set (*n* = 140, Additional file 1: Table S11) and a validation set (*n* = 35, Additional file 1: Table S12). Equation 6 was derived using the training set of 140 compounds.

6 $$log K_{p} = - 5.46 \left( { \pm 0.09} \right) + 0.47\left( { \pm 0.03} \right)log K_{ow } + 0.13 \left( { \pm 0.008} \right)log K_{aw }$$

$$n = 140,{ }R^{2} = 0.82,{\text{ Adj}}.{ }R^{2} = 0.82,{ }Q^{2} = 0.81,{ }RMSE = 0.47$$

$$PRESS{ }RMSE = 0.47, \; n_{external} = 35,{ }R_{external}^{2} = 0.81, RMSE_{external} = 0.48$$.

The fitting coefficients and regression statistics of Eq. 6 are statistically similar to Eq. 5. Predictions of Eq. 6 compared favorably with the experimental data for the external validation set ($R_{external}^{2} = 0.81, RMSE_{external} = 0.48$) (Additional file 1: Figure S4). The cross-validation statistics of Eq. 6 (Additional file 1: Table S13) are also similar to those of Eq. 5 (Additional file 1: Table S9). Being trained on full dataset (n = 175), users are recommended to prefer Eq. 5 over Eq. 6, which was trained on smaller dataset (n = 140).

When trained solely on the available experimental $K_{ow}$ and $K_{aw}$ data (*n* = 68), PPM (Additional file 1: Equation S2-1) yielded slightly better regression statistics ($n = 68,{ }R^{2} = 0.849,{\text{ Adj}}.{ }R^{2} = 0.844,{ }Q^{2} = 0.834,{ }RMSE = 0.339$) compared to those of Eq. 5, which was trained on ASM estimated values of $log K_{ow}$ and $log K_{aw}$. However, the regression statistics worsened ($n = 175,{ }R^{2} = 0.712,{\text{ Adj}}.{ }R^{2} = 0.708,{ }Q^{2} = 0.702,{ }RMSE = 0.589$) when the PPM is trained on the EPI-Suite (KOWWIN v1.69 and Henrywin v3.21) estimated values of $log K_{ow}$ and $log K_{aw}$ (Additional file 1: Equation S2-2). Model equations are further discussed in Additional file 1: Section S2. We recommend users to prefer Eq. 5 over Additional file 1: Equations S2-1, S2-2 for being trained on the larger and more accurate dataset.

Finally, we compared the explanatory power of $K_{ow}$ and $K_{aw}$ with that of other common physicochemical properties for describing the variance in $K_{p}$ data. When stepwise regression algorithm was applied on all descriptors ($K_{ow}$, $K_{aw}$, $K_{ocw}$, $BCF$, $D_{w}$ and $D_{ethanol}$), as the explanatory variables of $K_{p}$, only $K_{ow}$ and $K_{aw}$ were retained as statistically significant variables (Additional file 1: Table S14). Two models, based on the linear combinations of $log K_{ow}$ and $log D_{w}$, and of $log K_{ow}$ and $log D_{ethanol}$, were identified with $R^{2} = 0.81$ and 0.79, and $RMSE = 0.47$ and 0.50, respectively (Additional file 1: Table S14). These models are not discussed further, since $D_{w}$ and $D_{ethanol}$ are not as widely accessible as are the $K_{ow}$ and $K_{aw}$.

The PPM shows that skin permeability increases with increase in $K_{ow}$ and $K_{aw}$. This is expected as octanol is considered as a good surrogate medium of lipid [29]. However, stratum corneum is not exclusively comprised of lipids but also contain structural proteins (keratins) among other biotic phases [39], which play an important role in permeability [40], especially for the compounds exhibiting significant hydrogen bonding interactions [41]. The octanol–water system is not as sensitive to hydrogen bonding interactions as is the air–water system. This is evident from Pearson’s correlations (Fig. 1d) of $log K_{aw}$ with *A* ($r = - 0.36$) and *B* ($r = - 0.91$), which are higher in magnitude than the ones observed for the $log K_{ow}$ with *A* ($r = - 0.11$) and *B* ($r = - 0.10$). Chemicals with higher value of $K_{aw}$ would be more volatile and less-soluble in water phase [29]. The magnitude of $K_{aw}$ increases with the increase in the dispersive interactions and decrease in polarity/polarizability, and hydrogen-bonding interactions [32]. Hence, the greater is the value of $K_{aw}$ of the chemicals, the faster is the skin absorption of chemicals. Taken together, the PPM model sheds light on the propensity of chemical permeability in terms of widely used partitioning properties.

#### GC × GC model

The GC × GC model (Eq. 7 and Fig. 2b) successfully explained the variance in the $log K_{p}$ data of nonpolar organic chemicals. Here, $log K_{p}$ values of training set were estimated using the Zhang model due to lack of experimental $K_{p}$ values (Additional file 1: Table S3).

7 $$log K_{p} = - 5.35 \left( { \pm 0.07} \right) + 0.58\left( { \pm 0.02} \right)u_{1} - 3.51 \left( { \pm 0.19} \right)u_{2}$$

$$n = 79,{ }R^{2} = 0.90,{\text{ Adj}}.{ }R^{2} = 0.89,{ }Q^{2} = 0.89{ }$$ $$RMSE = 0.23,{ }PRESS{ }RMSE = 0.24$$

Due to lack of experimental values, the performance of Eq. 7 was evaluated by comparing its predicted values to those obtained by Zhang model and DERMWIN™. The RMSE shown for Eq. 7 is calculated by comparing Eq. 7’s predicted values of $log K_{p}$ with the Zhang model-predicted values. For the same model set, DERMWIN™ exhibited an RMSE of 0.93 *log* unit. The comparison of experimental values of $log K_{p}$, which were available only for 7 nonpolar chemicals, with Eq. 7’s predicted values yielded an RMSE of 0.48 *log* unit, which is in the neighborhood of the estimation error of Zhang Model (RMSE = 0.43).

For external validation, the full dataset of model nonpolar chemicals (*n* = 79) was split randomly with a ratio of 1:4 into a training set (*n* = 64, Additional file 1: Table S15) and an external validation set (*n* = 15, Additional file 1: Table S16). Equation 8 was derived using the training set of 64 compounds.

8 $$log K_{p} = - 5.34 \left( { \pm 0.08} \right) + 0.58\left( { \pm 0.03} \right)u_{1} - 3.56 \left( { \pm 0.22} \right)u_{2}$$

$$n = 64,{ }R^{2} = 0.89,{\text{ Adj}}.{ }R^{2} = 0.89,{ }Q^{2} = 0.89,{ }RMSE = 0.24{ }$$

$$PRESS{ }RMSE = 0.25, \; n_{external} = 15,{ }R_{external}^{2} = 0.85, RMSE_{external} = 0.22$$.

The fitting coefficients and regression statistics of Eq. 8 are statistically similar to Eq. 7. There was a good agreement (Additional file 1: Figure S5) between predictions of Eq. 8 and the predictions of the Zhang model (Eq. 2) for external validation set ($R_{external}^{2} = 0.85, RMSE_{external} = 0.22$).

Since the external validation approach can be sensitive to the partitioning of data into training set and validation set for the small datasets [42, 43] such as the GC × GC model set (*n* = 79), we performed four independent cross-validation tests on Eq. 7, which indicated that the GC × GC model is valid for predictive purpose (Additional file 1: Table S8). The cross-validation statistics of Eq. 8 (Additional file 1: Table S16) are also similar to those of Eq. 7 (Additional file 1: Table S8). Being trained on full dataset (n = 79), users are recommended to prefer Eq. 7 over Eq. 8, which was trained on smaller dataset (n = 64).

Finally, we validated the GC × GC model using the following independent approach. The experimental retention parameters, $u_{1}$ and $u_{2}$—obtained by analyzing 52 nonpolar chemicals on GC × GC instrument in a previous study [26]—were inputted in Eq. 7 to calculate $K_{p}$ values of nonpolar analytes. The calculated $K_{p}$ values by this means compared favorably with the $K_{p}$ values estimated by the Zhang model with $RMSE = 0.39$ (Additional file 1: Table S4).

By the virtue of Eq. 7, analysts can overlay the estimates of skin permeability coefficients on the GC × GC chromatograms of complex mixtures of nonpolar chemicals—akin to cases shown previously for the GC × GC chromatogram of polychlorinated alkane mixtures having several thousand congeners [26, 27].

## Limitations and outlook

The PPM model developed here works only for the neutral organic molecules. The model is not appropriate for the ionized species, which follows different partitioning [29] and permeation [44] behavior than is shown by neutral species. The PPM model can work only under the conditions where the permeants, if they have general acidic or basic functional groups such as carboxylic acids, phenols, or amines, are neutral. However, the partitioning behavior of ionized species may sufficiently be accounted for by considering descriptors such as pK*a* (acid dissociation constant) at a given pH of the system of interest [45]. Inclusion of the descriptors of ionizability in the model might extend the domain of its applicability to ionized species, which may be evaluated in a future study.

The GC × GC model, in its current form, is calibrated only for nonpolar chemicals, and is not considered suitable for the polar contaminants. This is because the combination of stationary phases (polydimethylsiloxane and phenylmethylpolysiloxane) used in developing the GC × GC model does not capture the hydrogen-bonding interactions adequately [46, 47]. However, the ionic liquid (IL) stationary phases may offer the opportunity to capture such interactions [48, 49], which may be evaluated in future studies to extend the application domain of the GC × GC model to polar contaminants.

The values of $log K_{ow}$ and $log K_{aw}$, used to train the PPM, were estimated using the ASM equations [30, 31] due to the scarcity of experimental data. Though the respective ASM equations are known to provide accurate estimates of $log K_{ow}$ and $log K_{aw}$ [30, 31], the predictive performance of the PPM is expected to improve if trained on the experimental data. In the same vein, the GC × GC Model, which is currently trained on the $log K_{p}$ values estimated by the Zhang model (Eq. 2), is expected to perform better when trained on the experimental data of $log K_{p}$. However, the training of models on the thin experimental data would lead to inflated errors around the regression coefficients for both models. The advantage of our approach is that we can estimate $K_{p}$ of neutral organic chemicals for which Abraham solute descriptors are not available.

In summary, the PPM performs better than the DERMWIN™ and similarly to the Zhang model. The DERMWIN™ model in EPI-Suite™ may be replaced easily with parsimonious PPM, as $K_{ow}$ and $K_{aw}$ values can be estimated with reasonable accuracy from EPI-Suite™. The GC × GC model predicts skin permeability of nonpolar chemicals with adequate accuracy, and can be applied to thousands of nonpolar analytes detected in complex environmental and technical mixtures. Thus, this study overcomes some of the limitations of existing models and illuminates a pathway for accurate and rapid risk assessment of neutral organic chemicals for their tendencies to penetrate human skin.

## Supplementary Information

**Additional file 1.** List of chemicals in the training/validation sets used for two-parameter model and GC × GC model with their values of experimental/estimated skin permeation coefficient and predictor variables; chemicals flagged to be outside of the application domain for the two models; Figure summarizing approach for the development of GC × GC model; cross-validation results of two models; and R script used to perform statistical tests.

## Data Availability

The additional information for this manuscript is available on the Springer Nature Publications website.
