# Supplementary material for: Development and evaluation of two-parameter linear free energy models for the prediction of human skin permeability coefficient of neutral organic chemicals
Source: J Cheminform. 2021 Mar 19;13:25. doi: 10.1186/s13321-021-00503-5 (PMC7980659; doi:10.1186/s13321-021-00503-5)
Supplement: Supplementary file 1 — Additional file 1. List of chemicals in the training/validation sets used for two-parameter model and GC × GC model with their values of experimental/estimated skin permeation coefficient and predictor variables; chemicals flagged to be outside of the application domain for the two models; Figure summarizing approach for the development of GC × GC model; cross-validation results of two models; and R script used to perform statistical tests. [file 13321_2021_503_MOESM1_ESM.docx]

Supplementary Information

Development and Evaluation of Two-Parameter Linear Free Energy Models for the Prediction of Human Skin Permeability Coefficient of Neutral Organic Chemicals

Sana Naseem^1^, Yasuyuki Zushi^2^, Deedar Nabi^1,3*^

^1^Institute of Environmental Sciences and Engineering (IESE), National University of Sciences and Technology (NUST), H-12, Islamabad, Pakistan.

^2^ Research Institute of Science for Safety and Sustainability, National Institute of Advanced Industrial Science and Technology (AIST), 16-1 Onogawa, Tsukuba, Ibaraki 305-8569, Japan

^3^College of Health Sciences, Jumeira University, Dubai, United Arab Emirates.

*deedar.nabi@iese.nust.edu.pk (corresponding author)

[Table S 1: Dataset showing experimental values of skin permeability coefficient, and Abraham solute descriptors used to develop 2-parameter partitioning model (PPM). 3](#_Toc64480240)

[Table S 2: Experimental and predicted values of octanol-water partition coefficient (Kow) and air-water partition coefficient (Kaw) for the PPM dataset. 11](#_Toc64480241)

[Table S 3: Model training set used to develop the GC×GC model. 19](#_Toc64480242)

[Table S 4: Validation set used to develop the GC×GC model. 23](#_Toc64480243)

[Table S 5: Chemicals flagged as influential in the PPM. 27](#_Toc64480244)

[Table S 6: Chemicals flagged as influential in the GC×GC model 28](#_Toc64480245)

[Table S 7: The PPM parameters with estimated standard error of beta – coefficients by bootstrapping test 29](#_Toc64480246)

[Table S 8: The GC ×GC Model parameters with estimated standard error of beta – coefficients by bootstrapping test 29](#_Toc64480247)

[Table S 9: Cross-Validation of the PPM using four independent test. 32](#_Toc64480248)

[Table S 10: Cross-Validation of the GC×GC Model using four independent test. 32](#_Toc64480249)

[Table S 11: Training Set for Equation 6. 33](#_Toc64480250)

[Table S 12: Validation Set for Equation 6. 39](#_Toc64480251)

[Table S 13: Cross-Validation of the PPM (equation 6) using four independent test. 42](#_Toc64480252)

[Table S 14: Selection of physicochemical variables for the estimation of skin permeability coefficients 44](#_Toc64480253)

[Table S 15: Training set for equation 8. 46](#_Toc64480254)

[Table S 16: Validation set for equation 8. 49](#_Toc64480255)

[Table S 17: Cross-Validation of the GCxGC (equation 6) using four independent test. 50](#_Toc64480256)

[Section S 1: Results of all cross validation tests for evaluation of model’s validity and robustness 30](#_Toc64480714)

[Section S 2: Training of Two - Parameter Partitioning Model (PPM) using EPI-Suite Estimated log $\boldsymbol{Kow}$ and log $\boldsymbol{Kaw}$ and using Experimental log $\boldsymbol{Kow}$ and log $\boldsymbol{Kaw}$ 43](#_Toc64480715)

[Section S 3:**:** R Code used to perform statistical analysis on the skin data 52](#_Toc64480716)

[Figure S 1: Overview of the methodology to develop the GC×GC Model 26](#_Toc33633876)

[Figure S 2: Influential plot for the PPM 27](#_Toc33633877)

[Figure S 3: Influence Plot for the GC×GC model 28](#_Toc33633878)

[Figure S 4: Linear regression plot for Two - Parameter Partitioning Model (PPM) showing training set and validation set.. 41](#_Toc33633879)

[Figure S 5: Linear regression plot for the GC×GC Model showing training set and validation set.. 51](#_Toc33633880)

Table S 1: Dataset showing experimental values of skin permeability coefficient, and Abraham solute descriptors used to develop 2-parameter partitioning model (PPM).

| SN | compounds | *E* | *S* | *A* | *B* | *V* | Exp$\log K_{p}$ | Pred$\log K_{p}$ by Zhang model |
| --- | --- | --- | --- | --- | --- | --- | --- | --- |
| 1 | 1-methoxy-2-propanol | 0.22 | 0.53 | 0.33 | 0.81 | 0.79 | -6.2 | -6.28 |
| 2 | 2,4,6-trichlorophenol | 1.01 | 0.8 | 0.6 | 0.15 | 1.14 | -4.3 | -4.19 |
| 3 | 2,4-dichlorophenol | 0.96 | 0.82 | 0.54 | 0.17 | 1.02 | -4.3 | -4.45 |
| 4 | 2-amino-4-nitrophenol | 1.42 | 1.95 | 1.01 | 0.43 | 1.05 | -6.54 | -5.81 |
| 5 | 2-chlorophenol | 0.85 | 0.88 | 0.32 | 0.31 | 0.9 | -4.56 | -4.99 |
| 6 | 2-ethoxyethanol | 0.24 | 0.55 | 0.29 | 0.82 | 0.79 | -6.68 | -6.3 |
| 7 | 2-hydroxypropyl nicotinate | 0.84 | 1.38 | 0.35 | 1.19 | 1.37 | -7.55 | -6.59 |
| 8 | 2-naphthol | 1.52 | 1.08 | 0.61 | 0.4 | 1.14 | -4.65 | -4.89 |
| 9 | 2-nitro-p-phenylenediamine | 1.53 | 2.05 | 0.35 | 0.7 | 1.09 | -6.66 | -6.22 |
| 10 | 2-phenylethanol | 0.81 | 0.82 | 0.31 | 0.66 | 1.06 | -5.2 | -5.52 |
| 11 | 3-nitrophenol | 1.05 | 1.57 | 0.79 | 0.23 | 0.95 | -5.33 | -5.25 |
| 12 | 4-amino-2-nitrophenol | 1.36 | 1.5 | 0.3 | 0.66 | 1.05 | -5.91 | -5.87 |
| 13 | 4-bromophenol | 1.08 | 1.17 | 0.67 | 0.2 | 0.95 | -4.52 | -4.87 |
| 14 | 4-butylphenol | 0.8 | 0.88 | 0.55 | 0.37 | 1.34 | -4.47 | -4.43 |
| 15 | 4-chloro-3,5-dimethylphenol | 0.93 | 0.96 | 0.64 | 0.21 | 1.18 | -4.31 | -4.48 |
| 16 | 4-chloro-3-methylphenol | 0.92 | 1.02 | 0.67 | 0.22 | 1.04 | -4.34 | -4.69 |
| 17 | 4-chloro-m-phenylenediamine | 1.36 | 1.5 | 0.23 | 0.69 | 1.04 | -6.54 | -5.93 |
| 18 | 4-chlorophenol | 0.92 | 1.08 | 0.67 | 0.2 | 0.9 | -4.52 | -4.95 |
| 19 | 4-cyanophenol | 0.94 | 1.63 | 0.8 | 0.29 | 0.93 | -5.53 | -5.49 |
| 20 | 4-ethylphenol | 0.8 | 0.9 | 0.55 | 0.36 | 1.06 | -4.53 | -4.92 |
| 21 | 4-hydroxybenzyl alcohol | 1 | 1.3 | 0.86 | 0.79 | 0.97 | -6.26 | -6.43 |
| 22 | 4-hydroxyphenylacetamide | 1.18 | 2.08 | 0.84 | 0.94 | 1.17 | -6.89 | -6.88 |
| 23 | 4-hydroxyphenylacetic acid | 1.03 | 1.45 | 0.94 | 0.74 | 1.13 | -6.16 | -6.14 |
| 24 | 4-nitrophenol | 1.07 | 1.72 | 0.82 | 0.26 | 0.95 | -5.33 | -5.42 |
| 25 | 4-propoxyphenol | 0.84 | 1.17 | 0.57 | 0.52 | 1.26 | -5.18 | -5.12 |
| 26 | 5,5-diethylbarbituric acid | 1.03 | 1.14 | 0.47 | 1.18 | 1.37 | -7.29 | -6.24 |
| 27 | 5-ethyl-5-(3-methylbutyl)barbital | 1.03 | 1.11 | 0.47 | 1.23 | 1.8 | -5.98 | -5.78 |
| 28 | 5-ethyl-5-butylbarbituric acid | 1.03 | 1.14 | 0.47 | 1.18 | 1.66 | -7.05 | -5.92 |
| 29 | 5-fluorouracil | 0.72 | 0.84 | 0.57 | 1.02 | 0.77 | -6.82 | -7.02 |
| 30 | 8-methoxypsoralen | 1.61 | 1.7 | 0 | 0.8 | 1.45 | -5.12 | -5.47 |
| 31 | acetic acid | 0.27 | 0.64 | 0.62 | 0.44 | 0.46 | -6.08 | -6.12 |
| 32 | aldosterone | 2.01 | 3.47 | 0.4 | 1.9 | 2.69 | -7.45 | -6.95 |
| 33 | alprenolol, | 1.25 | 1.09 | 0.15 | 1.44 | 2.16 | -5.3 | -5.48 |
| 34 | aniline | 0.96 | 0.96 | 0.26 | 0.41 | 0.82 | -4.94 | -5.39 |
| 35 | atenolol | 1.45 | 1.9 | 0.62 | 2.03 | 2.18 | -7.35 | -7.5 |
| 36 | benzaldehyde | 0.82 | 1 | 0 | 0.39 | 0.87 | -4.51 | -5.2 |
| 37 | benzene | 0.61 | 0.52 | 0 | 0.14 | 0.72 | -4.27 | -4.61 |
| 38 | benzoic acid | 0.73 | 0.9 | 0.59 | 0.4 | 0.93 | -5.68 | -5.27 |
| 39 | benzyl alcohol | 0.8 | 0.87 | 0.39 | 0.56 | 0.92 | -5.3 | -5.59 |
| 40 | benzyl nicotinate | 1.26 | 1.6 | 0 | 0.8 | 1.64 | -4.87 | -5.12 |
| 41 | bromoacetic acid | 0.56 | 1.06 | 0.77 | 0.38 | 0.64 | -6.01 | -5.92 |
| 42 | bromodichloromethane | 0.59 | 0.69 | 0.1 | 0.04 | 0.67 | -4.41 | -4.59 |
| 43 | butan-1-ol | 0.22 | 0.42 | 0.37 | 0.48 | 0.73 | -5.7 | -5.53 |
| 44 | butanoic acid | 0.21 | 0.64 | 0.61 | 0.45 | 0.75 | -6.41 | -5.64 |
| 45 | butyl nicotinate | 0.66 | 1.07 | 0 | 0.73 | 1.45 | -4.86 | -5.04 |
| 46 | butyl p-aminobenzoate | 1.01 | 1.35 | 0.3 | 0.68 | 1.6 | -4.41 | -4.89 |
| 47 | butyl paraben | 0.9 | 1.47 | 0.74 | 0.43 | 1.55 | -4.34 | -4.59 |
| 48 | 3, phenylproponic acid | 0.75 | 1.18 | 0.6 | 0.6 | 1.21 | -4.93 | -5.42 |
| 49 | 4-phenylbutanoic acid | 0.76 | 1.29 | 0.61 | 0.57 | 1.35 | -4.85 | -5.16 |
| 50 | 5-phenylpentanoic acid | 0.77 | 1.24 | 0.57 | 0.6 | 1.49 | -4.3 | -4.93 |
| 51 | 7-phenylheptanoic acid | 0.79 | 1.27 | 0.57 | 0.62 | 1.78 | -3.86 | -4.49 |
| 52 | benzoic acid | 0.73 | 0.9 | 0.59 | 0.4 | 0.93 | -4.91 | -5.27 |
| 53 | caffeine | 1.5 | 1.82 | 0.08 | 1.25 | 1.36 | -6.85 | -6.83 |
| 54 | catechol | 0.97 | 1.1 | 0.88 | 0.47 | 0.83 | -5.87 | -5.8 |
| 55 | chloral hydrate | 0.81 | 0.96 | 0.59 | 0.56 | 0.88 | -5.97 | -5.78 |
| 56 | chloroacetic acid, | 0.43 | 1.03 | 0.79 | 0.35 | 0.59 | -5.97 | -5.95 |
| 57 | chloroacetonitrile | 0.37 | 0.89 | 0.38 | 0.26 | 0.53 | -4.56 | -5.63 |
| 58 | chlorodibromomethane | 0.78 | 0.68 | 0.12 | 0.1 | 0.72 | -4.37 | -4.62 |
| 59 | chlorpheniramine | 1.47 | 1.41 | 0 | 1.33 | 2.21 | -5.93 | -5.24 |
| 60 | codeine | 2.22 | 2.15 | 0.15 | 1.8 | 2.21 | -6.57 | -6.74 |
| 61 | cortexolone | 1.91 | 3.45 | 0.36 | 1.6 | 2.74 | -7.2 | -6.23 |
| 62 | corticosterone | 1.86 | 3.43 | 0.4 | 1.63 | 2.74 | -6.84 | -6.32 |
| 63 | cortisone | 1.96 | 3.5 | 0.36 | 1.87 | 2.75 | -7.38 | -6.89 |
| 64 | decan-1-ol | 0.19 | 0.42 | 0.37 | 0.48 | 1.58 | -4.15 | -4.01 |
| 65 | dexamethasone | 2.04 | 3.51 | 0.71 | 1.92 | 2.91 | -7.27 | -6.84 |
| 66 | dibromoacetic acid, | 0.8 | 1.24 | 0.92 | 0.31 | 0.81 | -5.57 | -5.57 |
| 67 | dichloroacetic acid, | 0.48 | 1.2 | 0.92 | 0.26 | 0.71 | -5.75 | -5.65 |
| 68 | diclofenac | 1.81 | 1.85 | 0.55 | 0.77 | 2.03 | -5.3 | -4.61 |
| 69 | diethylcarbamazine | 0.65 | 1.3 | 0 | 1.55 | 1.72 | -6.15 | -6.69 |
| 70 | diethylether | 0.04 | 0.25 | 0 | 0.45 | 0.73 | -5.37 | -5.25 |
| 71 | dihydrocodeine | 2.06 | 2.32 | 0.26 | 1.74 | 2.25 | -6 | -6.51 |
| 72 | dimethylethylamine | 0.09 | 0.18 | 0 | 0.64 | 0.77 | -5.8 | -5.59 |
| 73 | ephedrine | 0.92 | 0.74 | 0.21 | 1.21 | 1.44 | -5.5 | -6.07 |
| 74 | estradiol | 1.8 | 1.77 | 0.86 | 1.1 | 2.2 | -5.61 | -5.16 |
| 75 | estrone | 1.73 | 2.05 | 0.5 | 1.08 | 2.16 | -5.52 | -5.25 |
| 76 | ethanol | 0.25 | 0.42 | 0.37 | 0.48 | 0.45 | -6.08 | -6.03 |
| 77 | ethyl nicotinate | 0.67 | 1.1 | 0 | 0.73 | 1.17 | -5.28 | -5.51 |
| 78 | ethyl p-aminobenzoate | 1.03 | 1.31 | 0.31 | 0.69 | 1.31 | -5.06 | -5.4 |
| 79 | ethylbenzene | 0.61 | 0.51 | 0 | 0.15 | 1 | -3 | -4.12 |
| 80 | etodolac | 1.8 | 2.17 | 1.05 | 1.15 | 2.24 | -5.48 | -5.52 |
| 81 | famotidine | 2.69 | 2.14 | 1.2 | 2.5 | 2.26 | -8.15 | -8.66 |
| 82 | fentanyl | 1.83 | 1.75 | 0 | 1.81 | 2.84 | -5.81 | -5.43 |
| 83 | fluocinonide | 1.95 | 2.48 | 0.31 | 2.51 | 3.46 | -6.33 | -6.54 |
| 84 | flurbiprofen, | 1.44 | 1.45 | 0.62 | 0.76 | 1.84 | -4.72 | -4.76 |
| 85 | glycerol trinitrate | 0.59 | 2.11 | 0 | 0.35 | 1.23 | -5.21 | -5.11 |
| 86 | griseofulvin | 1.75 | 2.64 | 0 | 1.44 | 2.39 | -6.44 | -5.88 |
| 87 | heptan-1-ol | 0.21 | 0.42 | 0.37 | 0.48 | 1.15 | -4.57 | -4.77 |
| 88 | heptanoic acid | 0.15 | 0.64 | 0.62 | 0.44 | 1.17 | -5.26 | -4.87 |
| 89 | hexan-1-ol | 0.21 | 0.42 | 0.37 | 0.48 | 1.01 | -4.92 | -5.02 |
| 90 | hexanoic acid | 0.17 | 0.63 | 0.62 | 0.44 | 1.03 | -5.48 | -5.12 |
| 91 | hexyl nicotinate | 0.63 | 1.07 | 0 | 0.73 | 1.74 | -4.83 | -4.54 |
| 92 | hydrocodone | 2.03 | 1.81 | 0 | 1.85 | 2.21 | -6.26 | -6.66 |
| 93 | hydrocortisone | 2.03 | 3.49 | 0.71 | 1.9 | 2.8 | -7.22 | -6.98 |
| 94 | hydrocortisone hydroxyhexanoate | 2.02 | 3.49 | 0.83 | 2.64 | 3.72 | -6.6 | -7.17 |
| 95 | hydrocortisone methylpimelate | 1.93 | 3.49 | 0.46 | 2.61 | 4.01 | -5.82 | -6.45 |
| 96 | hydromorphone | 2.04 | 1.6 | 0.16 | 1.95 | 2.06 | -7.83 | -6.72 |
| 97 | hydroquinone | 1.06 | 1.27 | 1.06 | 0.57 | 0.83 | -6.31 | -6.19 |
| 98 | hydroxypregnenolone | 1.55 | 3.35 | 0.57 | 1.35 | 2.72 | -6.3 | -5.72 |
| 99 | ibuprofen, neutral, | 0.73 | 0.7 | 0.57 | 0.79 | 1.78 | -4.58 | -4.57 |
| 100 | indomethacin | 2.24 | 1.47 | 0.58 | 1.43 | 2.53 | -5.39 | -5.03 |
| 101 | isoquinoline | 1.21 | 1 | 0 | 0.54 | 1.04 | -5.11 | -5.2 |
| 102 | ketoprofen, | 1.65 | 2.26 | 0.55 | 0.89 | 1.98 | -5.22 | -5.26 |
| 103 | ketorolac | 1.6 | 2.03 | 0.65 | 1.05 | 1.87 | -5.6 | -5.74 |
| 104 | lidocaine | 1.11 | 1.51 | 0.07 | 1.24 | 2.06 | -5.51 | -5.42 |
| 105 | mannitol | 0.84 | 2.33 | 0.87 | 1.77 | 1.31 | -8.42 | -8.53 |
| 106 | m-cresol | 0.82 | 0.88 | 0.57 | 0.34 | 0.92 | -4.89 | -5.12 |
| 107 | methanol | 0.28 | 0.44 | 0.43 | 0.47 | 0.31 | -6.38 | -6.29 |
| 108 | methyl 4-hydroxyphenylacetate | 0.91 | 1.36 | 0.59 | 0.7 | 1.27 | -5.26 | -5.64 |
| 109 | methyl p-aminobenzoate | 1.03 | 1.25 | 0.3 | 0.72 | 1.17 | -4.99 | -5.68 |
| 110 | methyl paraben | 0.93 | 1.46 | 0.71 | 0.46 | 1.13 | -5.03 | -5.41 |
| 111 | methylphenylether | 0.71 | 0.75 | 0 | 0.29 | 0.92 | -4.68 | -4.74 |
| 112 | methyltriglycol nicotinate | 0.73 | 1.42 | 0 | 1.79 | 2.05 | -6.83 | -6.74 |
| 113 | morphine | 2.23 | 1.3 | 0.39 | 2.01 | 2.06 | -7.24 | -7.1 |
| 114 | naproxen | 1.51 | 2.02 | 0.6 | 0.67 | 1.78 | -4.97 | -4.97 |
| 115 | nicotine | 0.87 | 0.88 | 0 | 1.09 | 1.37 | -5.34 | -5.92 |
| 116 | nimesulide | 2.11 | 2.7 | 0.31 | 1.09 | 2.08 | -6.35 | -5.69 |
| 117 | nizatidine | 1.91 | 2.92 | 0.64 | 2.41 | 2.46 | -7.78 | -8.09 |
| 118 | nonan-1-ol | 0.19 | 0.42 | 0.37 | 0.48 | 1.44 | -4.3 | -4.27 |
| 119 | o-cresol | 0.84 | 0.86 | 0.52 | 0.3 | 0.92 | -4.88 | -4.99 |
| 120 | octan-1-ol | 0.2 | 0.42 | 0.37 | 0.48 | 1.29 | -4.3 | -4.52 |
| 121 | octanoic acid | 0.15 | 0.65 | 0.62 | 0.45 | 1.31 | -5.18 | -4.65 |
| 122 | o-phenylenediamine | 1.26 | 1.4 | 0.24 | 0.73 | 0.92 | -6.7 | -6.21 |
| 123 | oxycodone | 2.32 | 2.5 | 0.29 | 1.91 | 2.26 | -6.43 | -7.64 |
| 124 | p-cresol | 0.82 | 0.87 | 0.57 | 0.31 | 0.92 | -4.83 | -5.04 |
| 125 | pentan-1-ol | 0.22 | 0.42 | 0.37 | 0.48 | 0.87 | -5.3 | -5.28 |
| 126 | pentanoic acid | 0.21 | 0.63 | 0.62 | 0.45 | 0.89 | -6.11 | -5.39 |
| 127 | phenobarbital | 1.63 | 1.72 | 0.71 | 1.18 | 1.7 | -6.7 | -6.19 |
| 128 | phenol | 0.81 | 0.89 | 0.6 | 0.3 | 0.78 | -5.27 | -5.29 |
| 129 | piroxicam, neutral | 2.56 | 2.9 | 0.17 | 1.49 | 2.25 | -6.02 | -6.6 |
| 130 | p-phenylenediamine | 1.3 | 1.66 | 0.44 | 0.83 | 0.92 | -6.98 | -6.67 |
| 131 | prednisolone | 2.21 | 3.1 | 0.71 | 1.92 | 2.75 | -7.91 | -6.85 |
| 132 | pregnenolone | 1.36 | 3.29 | 0.32 | 1.18 | 2.66 | -5.9 | -5.31 |
| 133 | progesterone | 1.45 | 3.29 | 0 | 1.14 | 2.62 | -4.9 | -5.17 |
| 134 | propan-1-ol | 0.24 | 0.42 | 0.37 | 0.48 | 0.59 | -5.93 | -5.78 |
| 135 | propranolol | 1.84 | 1.43 | 0.44 | 1.31 | 2.15 | -6.05 | -5.41 |
| 136 | ranitidine | 1.6 | 1.63 | 0.25 | 2.33 | 2.4 | -7.41 | -7.52 |
| 137 | resorcinol | 0.98 | 1.11 | 1.09 | 0.52 | 0.83 | -6.7 | -6 |
| 138 | scopolamine | 1.69 | 1.32 | 0.09 | 2.17 | 2.23 | -7.58 | -7.18 |
| 139 | sufentanil | 1.8 | 2.28 | 0 | 1.91 | 3.11 | -5.53 | -5.52 |
| 140 | testosterone | 1.54 | 2.56 | 0.32 | 1.17 | 2.38 | -5.54 | -5.33 |
| 141 | theophylline | 1.5 | 1.6 | 0.54 | 1.34 | 1.22 | -6.92 | -7.33 |
| 142 | thymol | 0.82 | 0.8 | 0.43 | 0.44 | 1.34 | -4.35 | -4.51 |
| 143 | toluene | 0.6 | 0.52 | 0 | 0.14 | 0.86 | -3.64 | -4.36 |
| 144 | tribromomethane | 0.97 | 0.68 | 0.15 | 0.06 | 0.77 | -4.34 | -4.41 |
| 145 | trichloroacetic acid, | 0.52 | 1.21 | 1.01 | 0.26 | 0.83 | -5.6 | -5.47 |
| 146 | trichloromethane | 0.43 | 0.49 | 0.15 | 0.02 | 0.62 | -4.46 | -4.56 |
| 147 | triglycol nicotinate | 0.95 | 1.58 | 0.37 | 1.78 | 1.91 | -8.08 | -7.16 |
| 148 | urea | 0.5 | 1.49 | 0.83 | 0.84 | 0.46 | -7.93 | -7.64 |
| 149 | water | 0 | 0.45 | 0.82 | 0.35 | 0.17 | -6.28 | -6.71 |
| 150 | 3,4-xylenol | 0.83 | 0.9 | 0.55 | 0.38 | 1.06 | -4.52 | -4.97 |
| 151 | 3-methylphenol | 0.82 | 0.88 | 0.57 | 0.34 | 0.92 | -4.89 | -5.12 |
| 152 | acetylsalicylic acid | 0.78 | 1.69 | 0.71 | 0.67 | 1.29 | -5.5 | -5.79 |
| 153 | aminopyrine | 1.68 | 1.74 | 0 | 1.6 | 1.87 | -6.55 | -6.68 |
| 154 | amylobarbital | 1.03 | 1.1 | 0.59 | 1.22 | 1.8 | -6 | -5.78 |
| 155 | anisole | 0.71 | 0.75 | 0 | 0.29 | 0.92 | -4.41 | -4.74 |
| 156 | barbital | 1.03 | 1 | 0.58 | 1.12 | 1.37 | -7.31 | -6.24 |
| 157 | benzenetriol | 1.18 | 1.37 | 1.4 | 0.68 | 0.89 | -6.98 | -6.51 |
| 158 | butan-2-one | 0.17 | 0.7 | 0 | 0.51 | 0.69 | -5.42 | -5.73 |
| 159 | butobarbital | 1.03 | 1.14 | 0.47 | 1.18 | 1.66 | -7.07 | -5.92 |
| 160 | butoxyethanol | 0.2 | 0.53 | 0.26 | 0.83 | 1.07 | -6 | -5.8 |
| 161 | coumarin | 1.23 | 1.68 | 0 | 0.52 | 1.06 | -5.6 | -5.53 |
| 162 | cyclobarbitone | 1.44 | 1.35 | 0.49 | 1.45 | 1.79 | -6.65 | -6.42 |
| 163 | ethylene glycol mono isopropyl ether | 0.2 | 0.48 | 0.21 | 0.91 | 0.93 | -6.71 | -6.2 |
| 164 | ethylene glycol mono methyl ether acetate | 0.17 | 0.79 | 0 | 0.81 | 0.95 | -6.1 | -6.05 |
| 165 | ethylene glycol mono propyl ether | 0.21 | 0.5 | 0.3 | 0.83 | 0.93 | -6.34 | -6.05 |
| 166 | iso-thymol | 0.82 | 0.81 | 0.56 | 0.43 | 1.34 | -4.84 | -4.53 |
| 167 | methyl nicotinate | 0.71 | 1.13 | 0 | 0.71 | 1.03 | -5.77 | -5.78 |
| 168 | methyl salicylate | 0.85 | 0.84 | 0.02 | 0.47 | 1.13 | -4.8 | -4.83 |
| 169 | o-tert-butylphenol | 0.82 | 0.92 | 0.52 | 0.4 | 1.34 | -4.48 | -4.51 |
| 170 | phloroglucinol | 1.36 | 1.39 | 1.4 | 0.73 | 0.89 | -5.87 | -6.62 |
| 171 | propylparaben | 0.9 | 1.45 | 0.74 | 0.43 | 1.41 | -5.41 | -4.84 |
| 172 | pyrogallol | 1.17 | 1.26 | 1.35 | 0.64 | 0.89 | -6.17 | -6.34 |
| 173 | digitoxin | 3.46 | 5.63 | 1.33 | 4.35 | 5.69 | -8.15 | -9.03 |
| 174 | ouabain | 4.01 | 6.2 | 0.9 | 3.46 | 4.16 | -9.66 | -9.75 |
| 175 | salicylic acid | 0.9 | 0.85 | 0.73 | 0.37 | 0.99 | -5.07 | -5.08 |

Table S 2: Experimental and predicted values of octanol-water partition coefficient (Kow) and air-water partition coefficient (Kaw) for the PPM dataset.

| SN | compounds | *log* $K_{ow}$  (exp) | *log* $K_{aw}$  (exp) | *log* $K_{ow}$  (ASM) | *log* $K_{aw}$  (ASM) | *log* $K_{ow}$  (KOWWIN) | *log* $K_{aw}$  (HenryWin) |
| --- | --- | --- | --- | --- | --- | --- | --- |
| 1 | 1-methoxy-2-propanol |  | -4.42 | -0.12 | -4.98 | -0.49 | -5.64 |
| 2 | 2,4,6-trichlorophenol | 3.69 | -3.97 | 3.67 | -3.65 | 3.45 | -5.03 |
| 3 | 2,4-dichlorophenol | 3.06 | -3.76 | 3.09 | -3.65 | 2.80 | -4.90 |
| 4 | 2-amino-4-nitrophenol | 1.26 |  | 1.39 | -9.82 | 0.99 | -10.50 |
| 5 | 2-chlorophenol | 2.15 | -3.34 | 2.00 | -3.69 | 2.16 | -4.77 |
| 6 | 2-ethoxyethanol | -0.32 | -4.72 | -0.17 | -4.94 | -0.42 | -5.64 |
| 7 | 2-hydroxypropyl nicotinate |  |  | 0.24 | -8.92 | 0.08 | -9.92 |
| 8 | 2-naphthol | 2.70 | -5.95 | 2.81 | -5.91 | 2.69 | -5.65 |
| 9 | 2-nitro-p-phenylenediamine | 0.53 |  | 0.54 | -8.90 | 0.55 | -8.62 |
| 10 | 2-phenylethanol | 1.36 | -4.98 | 1.44 | -5.03 | 1.57 | -4.93 |
| 11 | 3-nitrophenol | 2.00 | -7.09 | 1.88 | -6.92 | 1.91 | -7.04 |
| 12 | 4-amino-2-nitrophenol | 0.96 |  | 1.01 | -7.05 | 0.64 | -7.00 |
| 13 | 4-bromophenol | 2.59 | -5.21 | 2.46 | -5.27 | 2.40 | -5.04 |
| 14 | 4-butylphenol | 3.65 |  | 3.46 | -4.44 | 3.53 | -4.23 |
| 15 | 4-chloro-3,5-dimethylphenol | 3.27 |  | 3.27 | -4.53 | 3.25 | -4.68 |
| 16 | 4-chloro-3-methylphenol | 3.10 | -4.00 | 2.79 | -4.78 | 2.70 | -4.73 |
| 17 | 4-chloro-m-phenylenediamine | 0.85 |  | 0.86 | -6.94 | 0.25 | -7.69 |
| 18 | 4-chlorophenol | 2.39 | -4.59 | 2.22 | -5.04 | 2.16 | -4.77 |
| 19 | 4-cyanophenol | 1.60 |  | 1.47 | -7.35 | 1.61 | -6.65 |
| 20 | 4-ethylphenol | 2.58 | -4.50 | 2.40 | -4.69 | 2.55 | -4.47 |
| 21 | 4-hydroxybenzyl alcohol | 0.25 |  | 0.30 | -9.16 | 0.60 | -9.03 |
| 22 | 4-hydroxyphenylacetamide | -0.09 |  | -0.19 | -11.73 | 0.07 | -11.42 |
| 23 | 4-hydroxyphenylacetic acid | 0.75 |  | 0.93 | -9.48 | 0.95 | -9.73 |
| 24 | 4-nitrophenol | 1.91 | -7.77 | 1.63 | -7.57 | 1.91 | -7.04 |
| 25 | 4-propoxyphenol | 2.33 |  | 2.34 | -6.08 | 2.58 | -5.62 |
| 26 | 5,5-diethylbarbituric acid | 0.65 |  | 1.00 | -8.59 | 0.60 | -10.83 |
| 27 | 5-ethyl-5-(3-methylbutyl)barbital | 2.07 |  | 2.16 | -9.00 | 2.00 | -10.46 |
| 28 | 5-ethyl-5-butylbarbituric acid | 1.73 |  | 1.72 | -8.58 | 1.59 | -10.58 |
| 29 | 5-fluorouracil | -0.89 |  | -0.97 | -8.01 | -0.81 | -8.17 |
| 30 | 8-methoxypsoralen | 2.00 |  | 1.97 | -6.89 | 2.14 | -5.79 |
| 31 | acetic acid | -0.17 | -5.39 | -0.16 | -4.89 | 0.09 | -4.65 |
| 32 | aldosterone |  |  | 1.52 | -17.35 | 0.50 | -10.98 |
| 33 | alprenolol, | 3.10 |  | 2.90 | -8.18 | 2.81 | -9.32 |
| 34 | aniline | 0.90 | -4.08 | 1.32 | -4.28 | 1.08 | -4.11 |
| 35 | atenolol | 0.16 |  | 0.21 | -14.99 | -0.03 | -16.25 |
| 36 | benzaldehyde | 1.48 | -2.96 | 1.48 | -3.16 | 1.71 | -3.26 |
| 37 | benzene | 2.13 | -0.64 | 2.13 | -0.74 | 1.99 | -0.66 |
| 38 | benzoic acid | 1.87 | -5.81 | 1.74 | -5.10 | 1.87 | -5.35 |
| 39 | benzyl alcohol | 1.10 | -4.86 | 1.19 | -5.09 | 1.08 | -5.05 |
| 40 | benzyl nicotinate | 2.40 |  | 2.60 | -6.27 | 2.35 | -6.82 |
| 41 | bromoacetic acid | 0.41 | -6.57 | 0.44 | -6.25 | 0.43 | -5.59 |
| 42 | bromodichloromethane | 2.00 | -1.06 | 2.11 | -1.10 | 1.61 | -1.37 |
| 43 | butan-1-ol | 0.88 | -3.44 | 0.91 | -3.31 | 0.84 | -3.39 |
| 44 | butanoic acid | 0.79 | -4.66 | 0.85 | -4.62 | 1.07 | -4.40 |
| 45 | butyl nicotinate | 2.27 |  | 2.36 | -4.39 | 2.11 | -5.36 |
| 46 | butyl p-aminobenzoate | 2.87 |  | 2.98 | -6.08 | 2.78 | -5.93 |
| 47 | butyl paraben | 3.57 |  | 3.51 | -6.83 | 3.47 | -6.46 |
| 48 | 3, phenylproponic acid | 1.84 |  | 1.84 | -6.59 | 2.29 | -5.62 |
| 49 | 4-phenylbutanoic acid | 2.42 |  | 2.37 | -6.64 | 2.78 | -5.50 |
| 50 | 5-phenylpentanoic acid | 2.94 |  | 2.87 | -6.39 | 3.27 | -5.38 |
| 51 | 7-phenylheptanoic acid | 3.63 |  | 3.85 | -6.33 | 4.25 | -5.13 |
| 52 | benzoic acid | 1.87 | -5.81 | 1.74 | -5.10 | 1.87 | -5.35 |
| 53 | caffeine | -0.07 |  | -0.10 | -9.69 | 0.16 | -8.83 |
| 54 | catechol | 0.88 | -7.31 | 1.06 | -7.28 | 1.03 | -8.62 |
| 55 | chloral hydrate | 0.99 |  | 0.95 | -6.12 | 0.98 | -6.63 |
| 56 | chloroacetic acid, | 0.22 | -6.42 | 0.30 | -6.08 | 0.34 | -5.10 |
| 57 | chloroacetonitrile | 0.45 |  | 0.48 | -3.74 | 0.11 | -3.35 |
| 58 | chlorodibromomethane | 2.16 | -1.49 | 2.22 | -1.51 | 1.70 | -1.85 |
| 59 | chlorpheniramine | 3.38 |  | 3.26 | -7.97 | 3.82 | -7.78 |
| 60 | codeine | 1.19 |  | 1.35 | -13.18 | 1.28 | -11.51 |
| 61 | cortexolone | 3.08 |  | 2.46 | -15.65 | 3.15 | -4.19 |
| 62 | corticosterone | 1.94 |  | 2.35 | -15.87 | 1.99 | -7.82 |
| 63 | cortisone | 1.47 |  | 1.56 | -17.10 | 1.81 | -7.61 |
| 64 | decan-1-ol | 4.57 | -2.88 | 4.12 | -2.55 | 3.79 | -2.65 |
| 65 | dexamethasone | 1.94 |  | 2.04 | -18.61 | 1.72 | -5.53 |
| 66 | dibromoacetic acid, |  | -6.74 | 1.30 | -6.93 | 0.70 | -6.53 |
| 67 | dichloroacetic acid, | 0.92 | -6.47 | 0.94 | -6.49 | 0.52 | -5.56 |
| 68 | diclofenac | 4.51 |  | 4.24 | -8.84 | 4.02 | -9.71 |
| 69 | diethylcarbamazine |  |  | 0.30 | -8.70 | 0.37 | -9.75 |
| 70 | diethylether | 0.89 | -1.30 | 1.08 | -1.21 | 1.05 | -1.21 |
| 71 | dihydrocodeine |  |  | 1.66 | -12.32 | 1.49 | -11.45 |
| 72 | dimethylethylamine | 0.70 |  | 0.68 | -1.95 | 0.53 | -2.70 |
| 73 | ephedrine | 1.13 |  | 1.14 | -6.84 | 0.68 | -8.45 |
| 74 | estradiol | 4.01 |  | 3.85 | -11.26 | 3.94 | -8.83 |
| 75 | estrone | 3.13 |  | 3.41 | -10.50 | 3.43 | -7.81 |
| 76 | ethanol | -0.31 | -3.69 | -0.15 | -3.57 | -0.14 | -3.63 |
| 77 | ethyl nicotinate | 1.32 |  | 1.34 | -4.62 | 1.13 | -5.61 |
| 78 | ethyl p-aminobenzoate | 1.86 |  | 1.92 | -6.33 | 1.80 | -6.18 |
| 79 | ethylbenzene | 3.15 | -0.49 | 3.18 | -0.52 | 3.03 | -0.49 |
| 80 | etodolac |  |  | 3.42 | -13.21 | 3.93 | -11.24 |
| 81 | famotidine | -0.64 |  | -0.63 | -20.73 | -0.65 | -21.65 |
| 82 | fentanyl | 4.05 |  | 3.85 | -10.82 | 3.89 | -9.42 |
| 83 | fluocinonide | 3.19 |  | 3.10 | -16.78 | 2.77 | -14.06 |
| 84 | flurbiprofen, | 4.16 |  | 3.78 | -7.98 | 3.81 | -6.67 |
| 85 | glycerol trinitrate | 1.62 | -5.45 | 1.74 | -5.07 | 1.51 | -6.98 |
| 86 | griseofulvin | 2.18 |  | 2.45 | -11.64 | 1.92 | -11.24 |
| 87 | heptan-1-ol | 2.62 | -3.11 | 2.52 | -2.93 | 2.31 | -3.02 |
| 88 | heptanoic acid | 2.42 | -4.58 | 2.46 | -4.20 | 2.54 | -4.03 |
| 89 | hexan-1-ol | 2.03 | -3.16 | 1.98 | -3.05 | 1.82 | -3.14 |
| 90 | hexanoic acid | 1.92 | -4.51 | 1.94 | -4.31 | 2.05 | -4.16 |
| 91 | hexyl nicotinate | 3.51 |  | 3.41 | -4.13 | 3.10 | -5.12 |
| 92 | hydrocodone |  |  | 1.19 | -12.19 | 2.16 | -9.58 |
| 93 | hydrocortisone | 1.61 |  | 1.68 | -18.55 | 1.62 | -5.63 |
| 94 | hydrocortisone hydroxyhexanoate | 2.79 |  | 2.63 | -21.79 | 2.86 | -10.54 |
| 95 | hydrocortisone methylpimelate | 3.70 |  | 3.81 | -19.92 | 3.86 | -11.72 |
| 96 | hydromorphone |  |  | 1.23 | -12.20 | 1.60 | -12.34 |
| 97 | hydroquinone | 0.59 | -8.71 | 0.59 | -8.94 | 1.03 | -8.62 |
| 98 | hydroxypregnenolone |  |  | 3.18 | -14.79 | 3.71 | -4.13 |
| 99 | ibuprofen, neutral, | 3.97 |  | 3.83 | -5.67 | 3.79 | -5.21 |
| 100 | indomethacin | 4.27 |  | 4.52 | -10.99 | 4.23 | -11.89 |
| 101 | isoquinoline | 2.08 |  | 1.83 | -3.97 | 2.14 | -4.55 |
| 102 | ketoprofen, | 3.12 |  | 3.13 | -10.41 | 3.00 | -9.06 |
| 103 | ketorolac |  |  | 2.38 | -11.05 | 2.32 | -10.86 |
| 104 | lidocaine | 2.44 |  | 2.69 | -7.98 | 1.66 | -8.27 |
| 105 | mannitol | -3.10 |  | -2.38 | -16.09 | -3.01 | -10.53 |
| 106 | m-cresol | 1.96 | -4.46 | 1.96 | -4.75 | 2.06 | -4.60 |
| 107 | methanol | -0.77 | -3.73 | -0.65 | -3.94 | -0.63 | -3.76 |
| 108 | methyl 4-hydroxyphenylacetate | 1.63 |  | 1.62 | -7.53 | 1.60 | -7.22 |
| 109 | methyl p-aminobenzoate | 1.37 |  | 1.34 | -6.40 | 1.31 | -6.30 |
| 110 | methyl paraben | 1.96 |  | 1.82 | -7.22 | 2.00 | -6.83 |
| 111 | methylphenylether | 2.11 | -1.70 | 2.19 | -1.94 | 2.07 | -1.88 |
| 112 | methyltriglycol nicotinate |  |  | 0.64 | -9.93 | -0.18 | -11.16 |
| 113 | morphine | 0.89 |  | 0.89 | -12.65 | 0.72 | -14.26 |
| 114 | naproxen | 3.18 |  | 3.31 | -9.02 | 3.10 | -7.86 |
| 115 | nicotine | 1.17 |  | 1.11 | -5.84 | 1.00 | -6.91 |
| 116 | nimesulide | 2.60 |  | 2.61 | -11.77 | 2.22 | -8.38 |
| 117 | nizatidine |  |  | -0.42 | -17.96 | -0.43 | -16.00 |
| 118 | nonan-1-ol | 3.77 | -2.90 | 3.58 | -2.68 | 3.30 | -2.77 |
| 119 | o-cresol | 1.95 | -4.31 | 2.13 | -4.33 | 2.06 | -4.60 |
| 120 | octan-1-ol | 3.00 | -3.00 | 3.05 | -2.80 | 2.81 | -2.90 |
| 121 | octanoic acid | 3.05 | -4.44 | 2.95 | -4.15 | 3.03 | -3.91 |
| 122 | o-phenylenediamine | 0.15 | -6.53 | 0.30 | -6.96 | 0.16 | -7.56 |
| 123 | oxycodone |  |  | 0.16 | -15.66 | 0.66 | -14.02 |
| 124 | p-cresol | 1.94 | -4.39 | 2.08 | -4.58 | 2.06 | -4.60 |
| 125 | pentan-1-ol | 1.51 | -3.27 | 1.45 | -3.18 | 1.33 | -3.26 |
| 126 | pentanoic acid | 1.39 | -4.71 | 1.39 | -4.51 | 1.56 | -4.28 |
| 127 | phenobarbital | 1.47 |  | 1.62 | -11.28 | 1.33 | -12.17 |
| 128 | phenol | 1.46 | -4.87 | 1.55 | -4.81 | 1.51 | -4.64 |
| 129 | piroxicam, neutral | 3.06 |  | 1.87 | -15.74 | 2.58 | -16.93 |
| 130 | p-phenylenediamine | -0.30 |  | -0.29 | -8.89 | -0.39 | -7.56 |
| 131 | prednisolone | 1.62 |  | 1.96 | -17.80 | 1.40 | -5.96 |
| 132 | pregnenolone | 4.22 |  | 3.49 | -12.80 | 3.89 | -6.32 |
| 133 | progesterone | 3.87 |  | 3.50 | -11.48 | 3.67 | -5.58 |
| 134 | propan-1-ol | 0.25 | -3.52 | 0.38 | -3.44 | 0.35 | -3.51 |
| 135 | propranolol | 3.48 |  | 3.30 | -9.87 | 2.60 | -10.49 |
| 136 | ranitidine | 0.27 |  | 0.37 | -14.24 | 0.29 | -12.85 |
| 137 | resorcinol | 0.80 | -8.39 | 0.89 | -8.35 | 1.03 | -8.62 |
| 138 | scopolamine | 0.98 |  | 0.66 | -12.26 | 0.39 | -13.86 |
| 139 | sufentanil | 3.95 |  | 3.94 | -12.41 | 3.62 | -11.50 |
| 140 | testosterone | 3.32 |  | 3.32 | -11.24 | 3.27 | -6.84 |
| 141 | theophylline | -0.04 |  | -0.71 | -11.44 | -0.39 | -10.16 |
| 142 | thymol | 3.30 | -4.70 | 3.31 | -4.13 | 3.52 | -4.31 |
| 143 | toluene | 2.73 | -0.57 | 2.66 | -0.62 | 2.54 | -0.61 |
| 144 | tribromomethane | 2.40 | -1.66 | 2.67 | -1.49 | 1.79 | -2.34 |
| 145 | trichloroacetic acid, | 1.33 | -6.26 | 1.42 | -6.78 | 1.44 | -6.01 |
| 146 | trichloromethane | 1.97 | -0.82 | 2.10 | -0.64 | 1.52 | -0.88 |
| 147 | triglycol nicotinate |  |  | 0.11 | -11.95 | -0.88 | -13.66 |
| 148 | urea | -2.11 | -10.15 | -2.30 | -9.92 | -1.56 | -7.83 |
| 149 | water | -1.38 |  | -1.48 | -4.87 | -1.38 | -6.46 |
| 150 | 3,4-xylenol | 2.23 | -4.77 | 2.34 | -4.80 | 2.61 | -4.55 |
| 151 | 3-methylphenol | 1.96 | -4.46 | 1.96 | -4.75 | 2.06 | -4.60 |
| 152 | acetylsalicylic acid | 1.19 | -7.27 | 1.37 | -8.60 | 1.13 | -7.27 |
| 153 | aminopyrine | 1.00 |  | 0.79 | -10.54 | 0.60 | -9.25 |
| 154 | amylobarbital | 2.07 |  | 2.16 | -9.00 | 2.00 | -10.46 |
| 155 | anisole | 2.11 | -1.70 | 2.19 | -1.94 | 2.07 | -1.88 |
| 156 | barbital | 0.65 |  | 1.00 | -8.59 | 0.60 | -10.83 |
| 157 | benzenetriol |  |  | 0.41 | -11.04 | 0.97 | -12.61 |
| 158 | butan-2-one | 0.29 | -2.63 | 0.31 | -2.76 | 0.26 | -2.57 |
| 159 | butobarbital | 1.73 |  | 1.72 | -8.58 | 1.59 | -10.58 |
| 160 | butoxyethanol | 0.83 | -4.18 | 0.87 | -4.55 | 0.57 | -5.40 |
| 161 | coumarin | 1.39 | -5.39 | 1.27 | -5.60 | 1.51 | -3.55 |
| 162 | cyclobarbitone | 1.77 |  | 1.29 | -10.62 | 2.30 | -10.68 |
| 163 | ethylene glycol mono isopropyl ether | 0.05 | -4.42 | 0.10 | -4.74 | 0.00 | -5.52 |
| 164 | ethylene glycol mono methyl ether acetate |  | -4.90 | 0.16 | -4.22 | 0.10 | -3.95 |
| 165 | ethylene glycol mono propyl ether |  |  | 0.37 | -4.76 | 0.08 | -5.52 |
| 166 | iso-thymol | 3.49 |  | 3.34 | -4.60 | 3.52 | -4.31 |
| 167 | methyl nicotinate | 0.83 |  | 0.78 | -4.84 | 0.64 | -5.73 |
| 168 | methyl salicylate | 2.55 | -2.40 | 2.37 | -3.01 | 2.60 | -3.73 |
| 169 | o-tert-butylphenol | 3.31 | -2.98 | 3.32 | -4.58 | 3.42 | -4.23 |
| 170 | phloroglucinol | 0.16 |  | 0.32 | -11.43 | 0.55 | -12.61 |
| 171 | propylparaben | 3.04 |  | 3.00 | -6.90 | 2.98 | -6.58 |
| 172 | pyrogallol |  |  | 0.66 | -10.37 | 0.97 | -12.61 |
| 173 | digitoxin |  |  | 2.83 | -36.54 | 2.04 | -23.28 |
| 174 | ouabain | -2.00 |  | -0.24 | -33.70 | -2.83 | -20.14 |
| 175 | salicylic acid | 2.26 | -6.52 | 2.22 | -5.41 | 2.24 | -6.24 |

Table S 3: Model training set used to develop the GC×GC model.

| SN | Chemicals | $u_{1}$ | $u_{2}$ | *A* | *B* | *S* | *E* | *V* | *L* | $\log K_{p}$ (Zhang Model) | $\log K_{p}$  (GC×GC model) |
| --- | --- | --- | --- | --- | --- | --- | --- | --- | --- | --- | --- |
| 1 | nonane | 1.89 | -0.35 | 0 | 0 | 0 | 0 | 1.38 | 4.18 | -2.85 | -3.02 |
| 2 | decane | 2.14 | -0.37 | 0 | 0 | 0 | 0 | 1.52 | 4.69 | -2.6 | -2.79 |
| 3 | undecane | 2.39 | -0.4 | 0 | 0 | 0 | 0 | 1.66 | 5.19 | -2.35 | -2.56 |
| 4 | dodecane | 2.64 | -0.42 | 0 | 0 | 0 | 0 | 1.8 | 5.7 | -2.09 | -2.33 |
| 5 | methylcyclopentane | 1.28 | -0.24 | 0 | 0 | 0.1 | 0.23 | 0.85 | 2.91 | -3.84 | -3.78 |
| 6 | cyclooctane | 1.99 | -0.3 | 0 | 0 | 0.1 | 0.41 | 1.13 | 4.33 | -3.31 | -3.15 |
| 7 | cyclododecane | 2.92 | -0.38 | 0 | 0 | 0.1 | 0.56 | 1.69 | 6.19 | -2.27 | -2.32 |
| 8 | cyclohexadiene | 1.33 | -0.14 | 0 | 0.14 | 0.3 | 0.52 | 0.76 | 2.92 | -4.41 | -4.1 |
| 9 | 1,5,9-cyclododecatriene | 2.92 | -0.21 | 0 | 0.2 | 0.44 | 0.76 | 1.56 | 6.06 | -3.17 | -2.9 |
| 10 | 3-methylcyclohexene | 1.51 | -0.21 | 0 | 0.1 | 0.2 | 0.36 | 0.94 | 3.32 | -3.95 | -3.75 |
| 11 | cyclonona-1,2-diene | 2.07 | -0.18 | 0 | 0.12 | 0.35 | 0.53 | 1.18 | 4.38 | -3.63 | -3.5 |
| 12 | fluoromethane | -0.1 | 0 | 0 | 0.09 | 0.35 | 0.07 | 0.27 | 0.06 | -5.27 | -5.42 |
| 13 | 1-fluorobutane | 0.75 | -0.08 | 0 | 0.15 | 0.35 | 0.02 | 0.69 | 1.75 | -4.66 | -4.63 |
| 14 | 1-fluoropentane | 1.09 | -0.11 | 0 | 0.16 | 0.35 | 0 | 0.83 | 2.44 | -4.43 | -4.32 |
| 15 | 1-fluorononane | 2.17 | -0.22 | 0 | 0.15 | 0.35 | -0.02 | 1.39 | 4.61 | -3.4 | -3.32 |
| 16 | tetrafluoromethane | -0.65 | -0.22 | 0 | 0 | -0.2 | -0.55 | 0.32 | -0.82 | -4.71 | -4.95 |
| 17 | sulfur hexafluoride | -0.31 | -0.26 | 0 | 0 | -0.2 | -0.6 | 0.46 | -0.12 | -4.46 | -4.62 |
| 18 | 1-chlorobutane | 1.24 | -0.1 | 0 | 0.1 | 0.4 | 0.21 | 0.79 | 2.72 | -4.36 | -4.29 |
| 19 | 1-chlorooctane | 2.23 | -0.19 | 0 | 0.09 | 0.4 | 0.19 | 1.36 | 4.71 | -3.32 | -3.38 |
| 20 | carbon tetrachloride | 1.3 | -0.1 | 0 | 0 | 0.38 | 0.46 | 0.74 | 2.82 | -4.17 | -4.25 |
| 21 | 1,1,2-trichloroethane | 1.6 | 0.03 | 0.13 | 0.13 | 0.68 | 0.5 | 0.76 | 3.29 | -4.67 | -4.51 |
| 22 | hexachloroethane | 2.3 | -0.05 | 0 | 0 | 0.68 | 0.68 | 1.12 | 4.72 | -3.62 | -3.84 |
| 23 | γ-HCH | 3.86 | 0.11 | 0 | 0.5 | 1.28 | 1.45 | 1.58 | 7.59 | -4.28 | -3.49 |
| 24 | 1,3-butadiene, 1,1,2,3,4,4-hexachloro- | 2.63 | -0.14 | 0 | 0.1 | 0.52 | 1.02 | 1.32 | 5.42 | -3.37 | -3.34 |
| 25 | 1,3-cyclopentadiene, 1,2,3,4,5,5-hexachloro- | 2.77 | -0.17 | 0 | 0.09 | 0.48 | 1.1 | 1.35 | 5.73 | -3.25 | -3.15 |
| 26 | enflurane | 0.76 | -0.06 | 0.12 | 0.13 | 0.4 | -0.23 | 0.8 | 1.75 | -4.52 | -4.71 |
| 27 | 1-bromobutane | 1.44 | -0.11 | 0 | 0.12 | 0.4 | 0.36 | 0.85 | 3.11 | -4.29 | -4.13 |
| 28 | 1-bromooctane | 2.45 | -0.21 | 0 | 0.12 | 0.4 | 0.34 | 1.41 | 5.14 | -3.28 | -3.2 |
| 29 | dibromomethane | 1.41 | 0.06 | 0.11 | 0.07 | 0.69 | 0.71 | 0.6 | 2.89 | -4.78 | -4.74 |
| 30 | tribromomethane | 1.86 | 0.03 | 0.15 | 0.06 | 0.68 | 0.97 | 0.77 | 3.78 | -4.41 | -4.36 |
| 31 | hexabromoethane | 3.51 | 0.08 | 0 | 0 | 1.09 | 1.8 | 1.44 | 6.93 | -3.15 | -3.57 |
| 32 | diiodomethane | 1.89 | 0.03 | 0.05 | 0.17 | 0.69 | 1.2 | 0.77 | 3.86 | -4.63 | -4.34 |
| 33 | 1,2-diiodethane | 2.13 | 0.04 | 0.04 | 0.08 | 0.68 | 2.08 | 0.91 | 4.3 | -4.03 | -4.24 |
| 34 | 1-iodohexane | 2.2 | -0.17 | 0 | 0.15 | 0.4 | 0.62 | 1.21 | 4.62 | -3.67 | -3.48 |
| 35 | iodononane | 3.02 | -0.25 | 0 | 0.15 | 0.4 | 0.6 | 1.63 | 6.27 | -2.91 | -2.72 |
| 36 | 1-iodobutane | 1.7 | -0.12 | 0 | 0.14 | 0.4 | 0.63 | 0.93 | 3.63 | -4.15 | -3.93 |
| 37 | benzene | 1.26 | -0.13 | 0 | 0.14 | 0.29 | 0.57 | 0.72 | 2.79 | -4.48 | -4.15 |
| 38 | toluene | 1.57 | -0.06 | 0 | 0.14 | 0.52 | 0.56 | 0.86 | 3.33 | -4.36 | -4.23 |
| 39 | benzene, propyl- | 2.02 | -0.11 | 0 | 0.15 | 0.5 | 0.6 | 1.14 | 4.23 | -3.86 | -3.8 |
| 40 | benzene, butyl- | 1.97 | -0.1 | 0 | 0.15 | 0.5 | 0.55 | 1.28 | 4.13 | -3.62 | -3.84 |
| 41 | benzene, pentyl- | 2.52 | -0.15 | 0 | 0.15 | 0.51 | 0.59 | 1.42 | 5.23 | -3.37 | -3.35 |
| 42 | benzene, octyl- | 3.25 | -0.23 | 0 | 0.15 | 0.48 | 0.58 | 1.84 | 6.71 | -2.59 | -2.63 |
| 43 | benzene, decyl- | 3.75 | -0.29 | 0 | 0.15 | 0.47 | 0.58 | 2.13 | 7.71 | -2.08 | -2.16 |
| 44 | fluorobenzene | 1.31 | -0.01 | 0 | 0.1 | 0.57 | 0.48 | 0.73 | 2.79 | -4.53 | -4.54 |
| 45 | 1,3-difluorobenzene | 1.31 | -0.01 | 0 | 0.06 | 0.58 | 0.37 | 0.75 | 2.78 | -4.42 | -4.54 |
| 46 | 1,4-difluorobenzene | 1.31 | 0 | 0 | 0.06 | 0.6 | 0.38 | 0.75 | 2.77 | -4.43 | -4.58 |
| 47 | 1,3,5-trifluorobenzene | 1.36 | -0.01 | 0 | 0 | 0.62 | 0.25 | 0.77 | 2.88 | -4.29 | -4.54 |
| 48 | 1,2,3,5-tetrafluorobenzene | 1.28 | -0.01 | 0 | 0 | 0.59 | 0.29 | 0.79 | 2.73 | -4.23 | -4.57 |
| 49 | benzene, 1,3-dichloro- | 2.16 | -0.02 | 0 | 0.02 | 0.69 | 0.95 | 0.96 | 4.41 | -3.93 | -4.03 |
| 50 | benzene, 1,4-dichloro- | 2.18 | 0 | 0 | 0.02 | 0.75 | 0.83 | 0.96 | 4.44 | -3.99 | -4.09 |
| 51 | benzene, 1,2-dichloro- | 2.23 | 0.01 | 0 | 0.04 | 0.77 | 0.96 | 0.96 | 4.52 | -4.03 | -4.1 |
| 52 | benzene, 1,2,4-trichloro- | 2.58 | -0.03 | 0 | 0 | 0.74 | 0.97 | 1.08 | 5.25 | -3.7 | -3.73 |
| 53 | benzene, hexachloro- | 3.84 | -0.02 | 0 | 0 | 0.99 | 1.49 | 1.45 | 7.66 | -3.11 | -3.05 |
| 54 | bromobenzene | 1.98 | 0.01 | 0 | 0.09 | 0.73 | 0.88 | 0.89 | 4.04 | -4.26 | -4.25 |
| 55 | 1,4-dibromobenzene | 2.65 | 0.02 | 0 | 0.04 | 0.86 | 1.15 | 1.07 | 5.32 | -3.87 | -3.89 |
| 56 | 1,3-dibromobenzene | 2.65 | 0.03 | 0 | 0.04 | 0.88 | 1.17 | 1.07 | 5.33 | -3.88 | -3.92 |
| 57 | 1,3,5-tribromobenzene | 3.18 | 0.06 | 0 | 0 | 1.02 | 1.45 | 1.24 | 6.31 | -3.51 | -3.71 |
| 58 | 1,2,3,5-tetrabromobenzene | 3.86 | 0.11 | 0 | 0 | 1.24 | 1.83 | 1.42 | 7.57 | -3.28 | -3.5 |
| 59 | iodobenzene | 2.23 | 0.05 | 0 | 0.12 | 0.82 | 1.19 | 0.97 | 4.5 | -4.2 | -4.21 |
| 60 | 1,3-diiodobenzene | 3.23 | 0.09 | 0 | 0.09 | 1.07 | 1.83 | 1.23 | 6.38 | -3.73 | -3.8 |
| 61 | 1,4-diiodobenzene | 3.18 | 0.13 | 0 | 0.06 | 1.15 | 1.8 | 1.23 | 6.26 | -3.71 | -3.96 |
| 62 | naphthalene | 2.58 | 0.06 | 0 | 0.2 | 0.94 | 1.07 | 1.09 | 5.16 | -4.28 | -4.06 |
| 63 | naphthalene, 1-methyl- | 2.9 | 0.03 | 0 | 0.2 | 0.9 | 1.34 | 1.23 | 5.8 | -3.97 | -3.75 |
| 64 | acenaphthylene | 3.14 | 0.13 | 0 | 0.2 | 1.14 | 1.75 | 1.22 | 6.18 | -4.07 | -3.98 |
| 65 | acenaphthene | 3.26 | 0.07 | 0 | 0.2 | 1.04 | 1.6 | 1.26 | 6.47 | -3.96 | -3.68 |
| 66 | dibenzofuran | 3.38 | 0.04 | 0 | 0.17 | 1.02 | 1.41 | 1.27 | 6.72 | -3.88 | -3.51 |
| 67 | fluorene | 3.49 | 0.05 | 0 | 0.2 | 1.06 | 1.59 | 1.36 | 6.92 | -3.8 | -3.5 |
| 68 | phenanthrene | 3.9 | 0.14 | 0 | 0.26 | 1.29 | 2.06 | 1.45 | 7.63 | -3.84 | -3.57 |
| 69 | pyrene | 4.6 | 0.3 | 0 | 0.29 | 1.71 | 2.81 | 1.59 | 8.83 | -3.83 | -3.73 |
| 70 | benz[a]anthracene | 5.33 | 0.24 | 0 | 0.33 | 1.7 | 2.99 | 1.82 | 10.29 | -3.47 | -3.07 |
| 71 | chrysene | 5.35 | 0.25 | 0 | 0.36 | 1.73 | 3.03 | 1.82 | 10.33 | -3.56 | -3.1 |
| 72 | PCB 28 | 4.23 | 0.12 | 0 | 0.06 | 1.35 | 1.75 | 1.6 | 8.28 | -3.15 | -3.32 |
| 73 | PCB 52 | 4.76 | 0.07 | 0 | 0 | 1.33 | 1.92 | 1.64 | 9.35 | -2.92 | -2.82 |
| 74 | PCB 101 | 4.52 | 0.16 | 0 | 0 | 1.47 | 2.06 | 1.76 | 8.8 | -2.77 | -3.29 |
| 75 | PCB 118 | 4.85 | 0.22 | 0 | 0 | 1.67 | 2.01 | 1.85 | 9.4 | -2.74 | -3.3 |
| 76 | PCB 138 | 5.25 | 0.16 | 0 | 0 | 1.61 | 2.2 | 1.88 | 10.22 | -2.61 | -2.86 |
| 77 | PCB 153 | 5.18 | 0.17 | 0 | 0 | 1.61 | 2.2 | 1.88 | 10.08 | -2.61 | -2.93 |
| 78 | PCB 180 | 5.62 | 0.2 | 0 | 0 | 1.75 | 2.34 | 2.01 | 10.89 | -2.46 | -2.78 |
| 79 | p,p'-DDE | 4.96 | 0.07 | 0.06 | 0.14 | 1.36 | 1.8 | 2.05 | 9.73 | -2.58 | -2.7 |

Table S 4: Validation set used to develop the GC×GC model.

| SN | Chemicals | $u_{1}$ | $u_{2}$ | *A* | *B* | *S* | *E* | *V* | *L* | $\log K_{p}$ (Zhang Model) | $\log K_{p}$  (GC×GC model) |
| --- | --- | --- | --- | --- | --- | --- | --- | --- | --- | --- | --- |
| 1 | nonane | 1.98 | -0.45 | 0 | 0 | 0 | 0 | 1.38 | 4.18 | -2.85 | -2.61 |
| 2 | decane | 2.21 | -0.45 | 0 | 0 | 0 | 0 | 1.52 | 4.69 | -2.6 | -2.47 |
| 3 | undecane | 2.44 | -0.46 | 0 | 0 | 0 | 0 | 1.66 | 5.19 | -2.35 | -2.32 |
| 4 | dodecane | 2.68 | -0.46 | 0 | 0 | 0 | 0 | 1.8 | 5.7 | -2.09 | -2.17 |
| 5 | tetradecane | 3.14 | -0.47 | 0 | 0 | 0 | 0 | 2.08 | 6.71 | -1.59 | -1.88 |
| 6 | hexadecane | 3.61 | -0.47 | 0 | 0 | 0 | 0 | 2.36 | 7.71 | -1.08 | -1.59 |
| 7 | 1,1,2,3,4,4-hexachloro-1,3-butadiene | 2.66 | -0.23 | 0 | 0 | 0.85 | 1.02 | 1.32 | 5.42 | -3.46 | -2.99 |
| 8 | benzene, propyl- | 2.06 | -0.18 | 0 | 0.15 | 0.5 | 0.6 | 1.14 | 4.23 | -3.87 | -3.53 |
| 9 | benzene, butyl- | 2.29 | -0.17 | 0 | 0.15 | 0.5 | 0.55 | 1.28 | 4.13 | -3.62 | -3.42 |
| 10 | benzene, pentyl- | 2.53 | -0.15 | 0 | 0.15 | 0.51 | 0.59 | 1.42 | 5.23 | -3.37 | -3.36 |
| 11 | benzene, hexyl- | 2.77 | -0.17 | 0 | 0.15 | 0.5 | 0.59 | 1.56 | 5.72 | -3.11 | -3.16 |
| 12 | naphthalene | 3.86 | 0.24 | 0 | 0.2 | 0.94 | 1.07 | 1.09 | 0 | -4.24 | -3.93 |
| 13 | naphthalene, 2-methyl- | 2.8 | 0.02 | 0 | 0.25 | 0.81 | 1.3 | 1.23 | 0 | -4.04 | -3.79 |
| 14 | naphthalene, 1-methyl- | 2.83 | 0.05 | 0 | 0.2 | 0.9 | 1.34 | 1.23 | 0 | -4.04 | -3.89 |
| 15 | acenaphthylene | 3.13 | 0.17 | 0 | 0.2 | 1.14 | 1.75 | 1.22 | 0 | -4.22 | -4.14 |
| 16 | acenaphthene | 3.21 | 0.14 | 0 | 0.2 | 1.04 | 1.6 | 1.26 | 0 | -4.02 | -3.98 |
| 17 | dibenzofuran | 3.29 | 0.12 | 0 | 0.17 | 1.02 | 1.41 | 1.27 | 0 | -3.92 | -3.86 |
| 18 | fluorene | 3.44 | 0.14 | 0 | 0.2 | 1.06 | 1.59 | 1.36 | 0 | -3.92 | -3.85 |
| 19 | phenanthrene | 3.86 | 0.24 | 0 | 0.26 | 1.29 | 2.06 | 1.45 | 0 | -3.92 | -3.93 |
| 20 | anthracene | 3.88 | 0.23 | 0 | 0.26 | 1.34 | 2.29 | 1.45 | 0 | -3.89 | -3.9 |
| 21 | fluoranthene | 4.48 | 0.28 | 0 | 0.2 | 1.53 | 2.38 | 1.59 | 0 | -3.67 | -3.73 |
| 22 | pyrene | 4.59 | 0.32 | 0 | 0.28 | 1.71 | 2.81 | 1.59 | 0 | -3.81 | -3.8 |
| 23 | benz[a]anthracene | 5.34 | 0.33 | 0 | 0.33 | 1.7 | 2.99 | 1.82 | 0 | -3.52 | -3.41 |
| 24 | chrysene | 5.35 | 0.36 | 0 | 0.36 | 1.73 | 3.03 | 1.82 | 0 | -3.56 | -3.49 |
| 25 | benzene, 1,3-dichloro- | 2.15 | -0.16 | 0 | 0.02 | 0.69 | 0.95 | 0.96 | 0 | -3.97 | -3.52 |
| 26 | benzene, 1,4-dichloro- | 2.17 | -0.11 | 0 | 0.02 | 0.75 | 0.83 | 0.96 | 0 | -3.99 | -3.69 |
| 27 | benzene, 1,2-dichloro- | 2.22 | -0.13 | 0 | 0.04 | 0.77 | 0.96 | 0.96 | 0 | -4.05 | -3.62 |
| 28 | benzene, 1,2,4-trichloro- | 2.54 | -0.08 | 0 | 0 | 0.74 | 0.97 | 1.08 | 0 | -3.74 | -3.58 |
| 29 | benzene, hexachloro- | 3.74 | 0.05 | 0 | 0 | 0.99 | 1.49 | 1.45 | 0 | -3.19 | -3.36 |
| 30 | PCB 28 | 4.1 | 0.1 | 0 | 0.06 | 1.35 | 1.75 | 1.6 | 0 | -3.21 | -3.31 |
| 31 | PCB 52 | 4.27 | 0.09 | 0 | 0 | 1.33 | 1.92 | 1.64 | 0 | -3.07 | -3.18 |
| 32 | PCB 101 | 4.67 | 0.09 | 0 | 0 | 1.47 | 2.06 | 1.76 | 0 | -2.86 | -2.94 |
| 33 | PCB 105 | 5.08 | 0.07 | 0 | 0 | 1.67 | 2.01 | 1.85 | 0 | -2.8 | -2.64 |
| 34 | PCB 118 | 4.95 | 0.1 | 0 | 0 | 1.67 | 2.01 | 1.85 | 0 | -2.79 | -2.81 |
| 35 | PCB 128 | 5.3 | 0.19 | 0 | 0 | 1.61 | 2.2 | 1.88 | 0 | -2.65 | -2.94 |
| 36 | PCB 138 | 5.19 | 0.14 | 0 | 0 | 1.61 | 2.2 | 1.88 | 0 | -2.65 | -2.81 |
| 37 | PCB 153 | 5.05 | 0.16 | 0 | 0 | 1.61 | 2.2 | 1.88 | 0 | -2.65 | -2.97 |
| 38 | PCB 156 | 5.44 | 0.15 | 0 | 0 | 1.83 | 2.14 | 1.97 | 0 | -2.58 | -2.7 |
| 39 | PCB 170 | 5.67 | 0.17 | 0 | 0 | 1.75 | 2.34 | 2.01 | 0 | -2.44 | -2.63 |
| 40 | PCB 180 | 5.55 | 0.11 | 0 | 0 | 1.75 | 2.34 | 2.01 | 0 | -2.44 | -2.5 |
| 41 | p,p'-DDE | 4.8 | 0.1 | 0.06 | 0.14 | 1.4 | 1.8 | 2.05 | 0.06 | -2.6 | -2.88 |
| 42 | α-HCH | 3.66 | 0.19 | 0 | 0.47 | 1.2 | 1.45 | 1.58 | 0 | -4.46 | -3.87 |
| 43 | γ-HCH | 3.8 | 0.25 | 0 | 0.5 | 1.28 | 1.45 | 1.58 | 0 | -4.49 | -4.01 |
| 44 | β-HCH | 3.72 | 0.37 | 0.12 | 0.58 | 1.18 | 1.45 | 1.58 | 0.12 | -4.5 | -4.48 |
| 45 | δ-HCH | 3.82 | 0.39 | 0 | 0.4 | 1.1 | 1.38 | 1.58 | 0 | -4.39 | -4.51 |
| 46 | heptachlor | 4.18 | 0.03 | 0 | 0.56 | 0.85 | 2.08 | 1.96 | 0 | -3.39 | -3.02 |
| 47 | γ-chlordane (trans) | 4.6 | 0.09 | 0 | 0.47 | 1.2 | 1.45 | 1.58 | 0 | -3.19 | -2.99 |
| 48 | aldrin | 4.33 | 0.02 | 0 | 0.42 | 0.95 | 2.07 | 2.01 | 0 | -3 | -2.91 |
| 49 | p,p'-DDT | 5.17 | 0.15 | 0 | 0.16 | 1.76 | 1.81 | 2.22 | 9.53 | -2.55 | -2.87 |
| 50 | 4-chlorodiphenyl ether | 3.48 | 0.08 | 0 | 0.4 | 1.36 | 1.35 | 1.51 | 7.02 | -3.39 | -3.62 |
| 51 | 4-bromodiphenyl ether (PBDE 3) | 3.69 | 0.14 | 0 | 0.4 | 1.44 | 1.53 | 1.56 | 7.58 | -3.39 | -3.7 |
| 52 | bis(2,4-dibromophenyl) ether (PDBE 47) | 5.51 | 0.27 | 0 | 0.34 | 1.45 | 2.45 | 2.08 | 10.66 | -5.33 | -3.09 |

Primary Model Training Set (n=79)

6 ASM Solute Parameters

E, S, V, A, B, L

(Table S3)

Validation Set

54 nonpolar analytes analyzed on GCxGC

Conversion

SVD

Input

Validation

Calibration

Predicted log Kp by Zhang Model

Predicted log Kp

Conversion of *t_1_* and *t_2_* to *u_1_* and *u_2_*

LFER for Human Skin Permeability Coefficient

Primary Model Training Set (n=79)

2 Orthogonal Solute Parameters

*u_1_* and *u_2_*

Figure S 1: Overview of the methodology to develop the GC×GC Model

Table S 5: Chemicals flagged as influential in the PPM.

| Chemicals | Studentized Residuals | Hat values | Cook’s Distance |
| --- | --- | --- | --- |
| hydromorphone | -2.73 | 0.01 | 0.02 |
| butobarbital | -2.74 | 0.01 | 0.01 |
| digitoxin | 2.47 | 0.18 | 0.44 |
| ouabain | 1.22 | 0.15 | 0.08 |


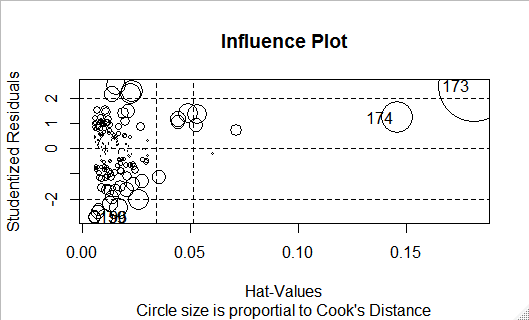


Figure S 2: Influential plot for the PPM

Table S 6: Chemicals flagged as influential in the GC×GC model

| Chemicals | Studentized Residuals | Hat values | Cook’s Distance |
| --- | --- | --- | --- |
| dodecane | 1.08 | 0.11 | 0.05 |
| cyclododecane | 0.23 | 0.10 | 0.00 |
| γ-HCH | -3.71 | 0.03 | 0.11 |
| chrysene | -2.09 | 0.08 | 0.12 |
| PCB 118 | 2.57 | 0.06 | 0.13 |


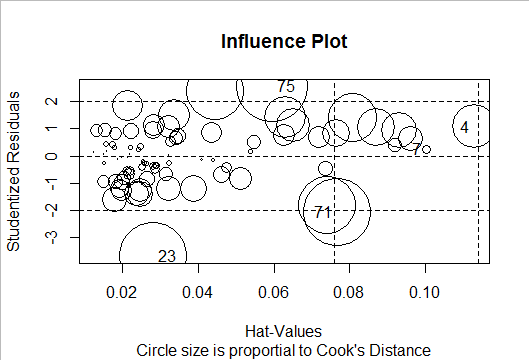


Figure S 3: Influence Plot for the GC×GC model

Table S 7: The PPM parameters with estimated standard error of beta – coefficients by bootstrapping test

| Parameter | Estimated Beta Coefficients | Std. Error | t value | Pr(>\|t\|) |
| --- | --- | --- | --- | --- |
| (Intercept) | -5.41 | 0.08 | -65.54 | **< 0.0001** |
| $\log K_{o-w}$ | 0.46 | 0.03 | 16.72 | **< 0.0001** |
| $\log K_{a-w}$ | 0.14 | 0.01 | 20.68 | **< 0.0001** |

Table S 8: The GC ×GC Model parameters with estimated standard error of beta – coefficients by bootstrapping test

| Parameter | Estimated Beta Coefficients | Std. Error | t value | Pr(>\|t\|) |
| --- | --- | --- | --- | --- |
| (Intercept) | -5.35 | 0.07 | -76.70 | **< 0.0001** |
| $u_{1}$ | 0.58 | 0.02 | 25.08 | **< 0.0001** |
| $u_{2}$ | -3.51 | 0.19 | -18.22 | **< 0.0001** |

Section S 1: Results of all cross validation tests for evaluation of model’s validity and robustness

1. **K – Nearest Neighbor (splitting of data into train and test data set):** In this technique, we split data into test and train sets by the ratio of 70:30, then regression applied. After this we asses various statistical property of test and train data in a comparative manners and nearness of indicators of train v/s test shows that model is robust and validated externally.
2. **Leave – One – Out Cross Validation**: This test involve the technique of abstracting one observation left out then training of the model will occur to check the result by removing one data point and error will be computed on held data set this test use for the assessment of internal validation.
3. **K – Fold Cross Validation**: It is a resampling technique In this test of validation occur on the splitting data chosen for validation Firstly mixing of data occur, then different k groups develop, then one group taken as test set and remaining sets consider as training sets, after all this process, model get trained on train sets and results of this training get evaluated on test data sets.
4. **Repeated K – Folds Cross Validation:** Algorithm develop for this technique based on the specific number of k , that validation of data sets will occur 10 times to ensure that after taking repeated folds, trained model is giving accurate results
5. **Bootstrapping:** This is a random resampling technique, in which different bootstraps develop by taking replicated samples taken from original sample .Then error of the model with overall summary of re - sampled model gets assessed.

Table S 9: Cross-Validation of the PPM using four independent test.

| **Indicators** | **KNN(splitting of data, test vs train)** | | **LOOCV** | **K - fold CV** | **Repeated K - fold CV** | | **CV by Bootstrapping** | | |
| --- | --- | --- | --- | --- | --- | --- | --- | --- | --- |
|  | KNN(test) | KNN(train) |  |  | 3 times | 10times | N= 100 | N=500 | N=1000 |
| **R2** | 0.87 | 0.81 | 0.81 | 0.84 | 0.83 | 0.83 | 0.81 | 0.81 | 0.81 |
| **RMSE** | 0.39 | 0.48 | 0.47 | 0.47 | 0.47 | 0.47 | 0.48 | 0.48 | 0.47 |
| **MAE** | 0.31 | 0.38 | 0.37 | 0.37 | 0.37 | 0.37 | 0.38 | 0.38 | 0.37 |
| **PRE^*^** | -0.07 | -0.08 |  |  |  |  |  |  |  |

*Prediction Error Rate = RMSE/Mean

Table S 10: Cross-Validation of the GC×GC Model using four independent test.

| **Indicators** | **KNN(splitting of data, test vs train)** | | **LOOCV** | **K - fold CV** | **Repeated K - fold CV** | | **CV by Bootstrapping** | | |
| --- | --- | --- | --- | --- | --- | --- | --- | --- | --- |
|  | KNN(test) | KNN(train) |  |  | 3 times | 10times | N= 100 | N=500 | N=1000 |
| **R2** | 0.86 | 0.91 | 0.89 | 0.91 | 0.90 | 0.90 | 0.89 | 0.89 | 0.89 |
| **RMSE** | 0.27 | 0.21 | 0.24 | 0.23 | 0.23 | 0.23 | 0.24 | 0.24 | 0.24 |
| **MAE** | 0.21 | 0.17 | 0.19 | 0.19 | 0.19 | 0.19 | 0.19 | 0.19 | 0.19 |
| **PRE*** | -0.07 | -0.06 |  |  |  |  |  |  |  |

Table S 11: Training Set for Equation 6.

| SN | Chemical | *log* $K_{ow}$ * | *log* $K_{aw}$ * | $\log K_{p}$  (exp) | $\log K_{p}$  (pred) |
| --- | --- | --- | --- | --- | --- |
| 1 | 1-methoxy-2-propanol | -0.12 | -4.98 | -6.20 | -6.19 |
| 2 | 2,4,6-trichlorophenol | 3.67 | -3.65 | -4.30 | -4.23 |
| 3 | 2,4-dichlorophenol | 3.09 | -3.65 | -4.30 | -4.51 |
| 4 | 2-amino-4-nitrophenol | 1.39 | -9.82 | -6.54 | -6.14 |
| 5 | 2-chlorophenol | 2.00 | -3.69 | -4.56 | -5.02 |
| 6 | 2-ethoxyethanol | -0.17 | -4.94 | -6.68 | -6.21 |
| 7 | 2-hydroxypropyl nicotinate | 0.24 | -8.92 | -7.55 | -6.55 |
| 8 | 2-naphthol | 2.81 | -5.91 | -4.65 | -4.94 |
| 9 | 2-phenylethanol | 1.44 | -5.03 | -5.20 | -5.46 |
| 10 | 3-nitrophenol | 1.88 | -6.92 | -5.33 | -5.51 |
| 11 | 4-amino-2-nitrophenol | 1.01 | -7.05 | -5.91 | -5.94 |
| 12 | 4-bromophenol | 2.46 | -5.27 | -4.52 | -5.02 |
| 13 | 4-chloro-3,5-dimethylphenol | 3.27 | -4.53 | -4.31 | -4.54 |
| 14 | 4-chloro-m-phenylenediamine | 0.86 | -6.94 | -6.54 | -5.99 |
| 15 | 4-chlorophenol | 2.22 | -5.04 | -4.52 | -5.10 |
| 16 | 4-ethylphenol | 2.40 | -4.69 | -4.53 | -4.97 |
| 17 | 4-hydroxybenzyl alcohol | 0.30 | -9.16 | -6.26 | -6.56 |
| 18 | 4-hydroxyphenylacetamide | -0.19 | -11.73 | -6.89 | -7.13 |
| 19 | 4-hydroxyphenylacetic acid | 0.93 | -9.48 | -6.16 | -6.30 |
| 20 | 4-nitrophenol | 1.63 | -7.57 | -5.33 | -5.72 |
| 21 | 4-propoxyphenol | 2.34 | -6.08 | -5.18 | -5.18 |
| 22 | 5-ethyl-5-(3-methylbutyl)barbital | 2.16 | -9.00 | -5.98 | -5.66 |
| 23 | 5-fluorouracil | -0.97 | -8.01 | -6.82 | -6.99 |
| 24 | 8-methoxypsoralen | 1.97 | -6.89 | -5.12 | -5.47 |
| 25 | aldosterone | 1.52 | -17.35 | -7.45 | -7.09 |
| 26 | alprenolol | 2.90 | -8.18 | -5.30 | -5.20 |
| 27 | atenolol | 0.21 | -14.99 | -7.35 | -7.39 |
| 28 | benzaldehyde | 1.48 | -3.16 | -4.51 | -5.19 |
| 29 | benzene | 2.13 | -0.74 | -4.27 | -4.56 |
| 30 | benzoic acid | 1.74 | -5.10 | -5.68 | -5.33 |
| 31 | benzyl alcohol | 1.19 | -5.09 | -5.30 | -5.59 |
| 32 | bromoacetic acid | 0.44 | -6.25 | -6.01 | -6.10 |
| 33 | bromodichloromethane | 2.11 | -1.10 | -4.41 | -4.62 |
| 34 | butan-1-ol | 0.91 | -3.31 | -5.70 | -5.48 |
| 35 | butanoic acid | 0.85 | -4.62 | -6.41 | -5.69 |
| 36 | butyl nicotinate | 2.36 | -4.39 | -4.86 | -4.95 |
| 37 | butyl p-aminobenzoate | 2.98 | -6.08 | -4.41 | -4.89 |
| 38 | butyl paraben | 3.51 | -6.83 | -4.34 | -4.74 |
| 39 | C6H5(CH2)3COOH | 2.37 | -6.64 | -4.85 | -5.24 |
| 40 | C6H5(CH2)4COOH | 2.87 | -6.39 | -4.30 | -4.98 |
| 41 | C6H5(CH2)7COOH | 3.85 | -6.33 | -3.86 | -4.51 |
| 42 | caffeine | -0.10 | -9.69 | -6.85 | -6.82 |
| 43 | chloral hydrate | 0.95 | -6.12 | -5.97 | -5.84 |
| 44 | chloroacetic acid | 0.30 | -6.08 | -5.97 | -6.14 |
| 45 | chloroacetonitrile | 0.48 | -3.74 | -4.56 | -5.74 |
| 46 | chlorpheniramine | 3.26 | -7.97 | -5.93 | -5.01 |
| 47 | cortexolone | 2.46 | -15.65 | -7.20 | -6.42 |
| 48 | corticosterone | 2.35 | -15.87 | -6.84 | -6.50 |
| 49 | cortisone | 1.56 | -17.10 | -7.38 | -7.04 |
| 50 | decan-1-ol | 4.12 | -2.55 | -4.15 | -3.88 |
| 51 | dibromoacetic acid, Fn = 0.15, log Kp(t) = 6.25 | 1.30 | -6.93 | -5.57 | -5.79 |
| 52 | dichloroacetic acid, Fn = 0.20, log Kp(t) = 6.39 | 0.94 | -6.49 | -5.75 | -5.90 |
| 53 | diclofenac | 4.24 | -8.84 | -5.30 | -4.67 |
| 54 | diethylcarbamazine | 0.30 | -8.70 | -6.15 | -6.49 |
| 55 | diethylether | 1.08 | -1.21 | -5.37 | -5.12 |
| 56 | dihydrocodeine | 1.66 | -12.32 | -6.00 | -6.34 |
| 57 | dimethylethylamine | 0.68 | -1.95 | -5.80 | -5.41 |
| 58 | ephedrine | 1.14 | -6.84 | -5.50 | -5.85 |
| 59 | ethanol | -0.15 | -3.57 | -6.08 | -6.01 |
| 60 | ethyl nicotinate | 1.34 | -4.62 | -5.28 | -5.46 |
| 61 | ethyl p-aminobenzoate | 1.92 | -6.33 | -5.06 | -5.41 |
| 62 | etodolac | 3.42 | -13.21 | -5.48 | -5.64 |
| 63 | flurbiprofen, neutral, Fn = 0.01, log | 3.78 | -7.98 | -4.72 | -4.77 |
| 64 | glycerol trinitrate | 1.74 | -5.07 | -5.21 | -5.33 |
| 65 | griseofulvin | 2.45 | -11.64 | -6.44 | -5.88 |
| 66 | hexan-1-ol | 1.98 | -3.05 | -4.92 | -4.95 |
| 67 | hexanoic acid | 1.94 | -4.31 | -5.48 | -5.13 |
| 68 | hexyl nicotinate | 3.41 | -4.13 | -4.83 | -4.42 |
| 69 | hydrocodone | 1.19 | -12.19 | -6.26 | -6.55 |
| 70 | hydrocortisone | 1.68 | -18.55 | -7.22 | -7.18 |
| 71 | hydrocortisone methylpimelate | 3.81 | -19.92 | -5.82 | -6.37 |
| 72 | hydromorphone | 1.23 | -12.20 | -7.83 | -6.53 |
| 73 | hydroquinone | 0.59 | -8.94 | -6.31 | -6.39 |
| 74 | hydroxypregnenolone | 3.18 | -14.79 | -6.30 | -5.97 |
| 75 | ibuprofen, neutral, Fn = 0.03, log Kp(t) = 5.83 | 3.83 | -5.67 | -4.58 | -4.43 |
| 76 | isoquinoline | 1.83 | -3.97 | -5.11 | -5.14 |
| 77 | ketoprofen | 3.13 | -10.41 | -5.22 | -5.40 |
| 78 | ketorolac | 2.38 | -11.05 | -5.60 | -5.84 |
| 79 | lidocaine | 2.69 | -7.98 | -5.51 | -5.28 |
| 80 | mannitol | -2.38 | -16.09 | -8.42 | -8.75 |
| 81 | m-cresol | 1.96 | -4.75 | -4.89 | -5.18 |
| 82 | methanol | -0.65 | -3.94 | -6.38 | -6.30 |
| 83 | methyl 4-hydroxyphenylacetate | 1.62 | -7.53 | -5.26 | -5.72 |
| 84 | methyl p-aminobenzoate | 1.34 | -6.40 | -4.99 | -5.69 |
| 85 | methyl paraben | 1.82 | -7.22 | -5.03 | -5.58 |
| 86 | methyltriglycol nicotinate | 0.64 | -9.93 | -6.83 | -6.50 |
| 87 | naproxen | 3.31 | -9.02 | -4.97 | -5.12 |
| 88 | nimesulide | 2.61 | -11.77 | -6.35 | -5.83 |
| 89 | nizatidine | -0.42 | -17.96 | -7.78 | -8.08 |
| 90 | nonan-1-ol | 3.58 | -2.68 | -4.30 | -4.14 |
| 91 | octan-1-ol | 3.05 | -2.80 | -4.30 | -4.41 |
| 92 | octanoic acid | 2.95 | -4.15 | -5.18 | -4.64 |
| 93 | o-phenylenediamine | 0.30 | -6.96 | -6.70 | -6.26 |
| 94 | oxycodone | 0.16 | -15.66 | -6.43 | -7.50 |
| 95 | p-cresol | 2.08 | -4.58 | -4.83 | -5.11 |
| 96 | pentanoic acid | 1.39 | -4.51 | -6.11 | -5.42 |
| 97 | phenobarbital | 1.62 | -11.28 | -6.70 | -6.22 |
| 98 | piroxicam, neutral | 1.87 | -15.74 | -6.02 | -6.71 |
| 99 | p-phenylenediamine | -0.29 | -8.89 | -6.98 | -6.80 |
| 100 | prednisolone | 1.96 | -17.80 | -7.91 | -6.94 |
| 101 | pregnenolone | 3.49 | -12.80 | -5.90 | -5.55 |
| 102 | progesterone | 3.50 | -11.48 | -4.90 | -5.37 |
| 103 | propranolol (Fn larger than 0.85) | 3.30 | -9.87 | -6.05 | -5.25 |
| 104 | ranitidine | 0.37 | -14.24 | -7.41 | -7.21 |
| 105 | resorcinol | 0.89 | -8.35 | -6.70 | -6.17 |
| 106 | scopolamine | 0.66 | -12.26 | -7.58 | -6.81 |
| 107 | sufentanil | 3.94 | -12.41 | -5.53 | -5.29 |
| 108 | theophylline | -0.71 | -11.44 | -6.92 | -7.34 |
| 109 | thymol | 3.31 | -4.13 | -4.35 | -4.47 |
| 110 | toluene | 2.66 | -0.62 | -3.64 | -4.30 |
| 111 | tribromomethane | 2.67 | -1.49 | -4.34 | -4.41 |
| 112 | trichloroacetic acid, Fn = 0.10, log Kp(t) = 6.39 | 1.42 | -6.78 | -5.60 | -5.71 |
| 113 | trichloromethane | 2.10 | -0.64 | -4.46 | -4.56 |
| 114 | triglycol nicotinate | 0.11 | -11.95 | -8.08 | -7.02 |
| 115 | urea | -2.30 | -9.92 | -7.93 | -7.88 |
| 116 | water | -1.48 | -4.87 | -6.28 | -6.81 |
| 117 | 3,4-xylenol | 2.34 | -4.80 | -4.52 | -5.01 |
| 118 | 3-methylphenol | 1.96 | -4.75 | -4.89 | -5.18 |
| 119 | acetylsalicylic acid | 1.37 | -8.60 | -5.50 | -5.98 |
| 120 | aminopyrine | 0.79 | -10.54 | -6.55 | -6.51 |
| 121 | amylobarbital | 2.16 | -9.00 | -6.00 | -5.66 |
| 122 | anisole | 2.19 | -1.94 | -4.41 | -4.70 |
| 123 | barbital | 1.00 | -8.59 | -7.31 | -6.15 |
| 124 | benzenetriol | 0.41 | -11.04 | -6.98 | -6.76 |
| 125 | butobarbital | 1.72 | -8.58 | -7.07 | -5.81 |
| 126 | butoxyethanol | 0.87 | -4.55 | -6.00 | -5.67 |
| 127 | coumarin | 1.27 | -5.60 | -5.60 | -5.62 |
| 128 | cyclobarbitone | 1.29 | -10.62 | -6.65 | -6.29 |
| 129 | ethylene glycol mono isopropyl ether | 0.10 | -4.74 | -6.71 | -6.05 |
| 130 | ethylene glycol mono methyl ether acetate | 0.16 | -4.22 | -6.10 | -5.96 |
| 131 | ethylene glycol mono propyl ether | 0.37 | -4.76 | -6.34 | -5.93 |
| 132 | methyl nicotinate | 0.78 | -4.84 | -5.77 | -5.75 |
| 133 | methyl salicylate | 2.37 | -3.01 | -4.80 | -4.76 |
| 134 | o-tert-butylphenol | 3.32 | -4.58 | -4.48 | -4.52 |
| 135 | phloroglucinol | 0.32 | -11.43 | -5.87 | -6.85 |
| 136 | propylparaben | 3.00 | -6.90 | -5.41 | -4.99 |
| 137 | pyrogallol | 0.66 | -10.37 | -6.17 | -6.55 |
| 138 | digitoxin | 2.83 | -36.54 | -8.15 | -9.06 |
| 139 | ouabain | -0.24 | -33.70 | -9.66 | -10.12 |
| 140 | salicylic acid | 2.22 | -5.41 | -5.07 | -5.15 |

* The values of *log* $K_{ow}$ and *log* $K_{aw}$ were estimated using respective Abraham Solvation Model equations

Table S 12: Validation Set for Equation 6.

| SN | Chemical | *log* $K_{ow}$ * | *log* $K_{aw}$ * | $\log K_{p}$  (exp) | $\log K_{p}$  (pred) |
| --- | --- | --- | --- | --- | --- |
| 1 | 2-nitro-p-phenylenediamine | 0.54 | -8.90 | -6.66 | -6.41 |
| 2 | 4-butylphenol | 3.46 | -4.44 | -4.47 | -4.44 |
| 3 | 4-chloro-3-methylphenol | 2.79 | -4.78 | -4.34 | -4.80 |
| 4 | 4-cyanophenol | 1.47 | -7.35 | -5.53 | -5.76 |
| 5 | 5,5-diethylbarbituric acid | 1.00 | -8.59 | -7.29 | -6.15 |
| 6 | 5-ethyl-5-butylbarbituric acid | 1.72 | -8.58 | -7.05 | -5.81 |
| 7 | acetic acid | -0.16 | -4.89 | -6.08 | -6.20 |
| 8 | aniline | 1.32 | -4.28 | -4.94 | -5.42 |
| 9 | benzyl nicotinate | 2.60 | -6.27 | -4.87 | -5.09 |
| 10 | C6H5(CH2)2COOH | 1.84 | -6.59 | -4.93 | -5.49 |
| 11 | C6H5COOH | 1.74 | -5.10 | -4.91 | -5.33 |
| 12 | catechol | 1.06 | -7.28 | -5.87 | -5.95 |
| 13 | chlorodibromomethane | 2.22 | -1.51 | -4.37 | -4.62 |
| 14 | codeine | 1.35 | -13.18 | -6.57 | -6.61 |
| 15 | dexamethasone | 2.04 | -18.61 | -7.27 | -7.02 |
| 16 | estradiol | 3.85 | -11.26 | -5.61 | -5.18 |
| 17 | estrone | 3.41 | -10.50 | -5.52 | -5.28 |
| 18 | ethylbenzene | 3.18 | -0.52 | -3.00 | -4.04 |
| 19 | famotidine | -0.63 | -20.73 | -8.15 | -8.55 |
| 20 | fentanyl | 3.85 | -10.82 | -5.81 | -5.12 |
| 21 | fluocinonide | 3.10 | -16.78 | -6.33 | -6.27 |
| 22 | heptan-1-ol | 2.52 | -2.93 | -4.57 | -4.68 |
| 23 | heptanoic acid | 2.46 | -4.20 | -5.26 | -4.88 |
| 24 | hydrocortisone hydroxyhexanoate | 2.63 | -21.79 | -6.60 | -7.17 |
| 25 | indomethacin | 4.52 | -10.99 | -5.39 | -4.82 |
| 26 | methylphenylether | 2.19 | -1.94 | -4.68 | -4.70 |
| 27 | morphine | 0.89 | -12.65 | -7.24 | -6.75 |
| 28 | nicotine | 1.11 | -5.84 | -5.34 | -5.73 |
| 29 | o-cresol | 2.13 | -4.33 | -4.88 | -5.05 |
| 30 | pentan-1-ol | 1.45 | -3.18 | -5.30 | -5.21 |
| 31 | phenol | 1.55 | -4.81 | -5.27 | -5.39 |
| 32 | propan-1-ol | 0.38 | -3.44 | -5.93 | -5.75 |
| 33 | testosterone | 3.32 | -11.24 | -5.54 | -5.42 |
| 34 | butan-2-one | 0.31 | -2.76 | -5.42 | -5.69 |
| 35 | iso-thymol | 3.34 | -4.60 | -4.84 | -4.52 |

* The values of *log* $K_{ow}$ and *log* $K_{aw}$ were estimated using respective Abraham Solvation Model equations


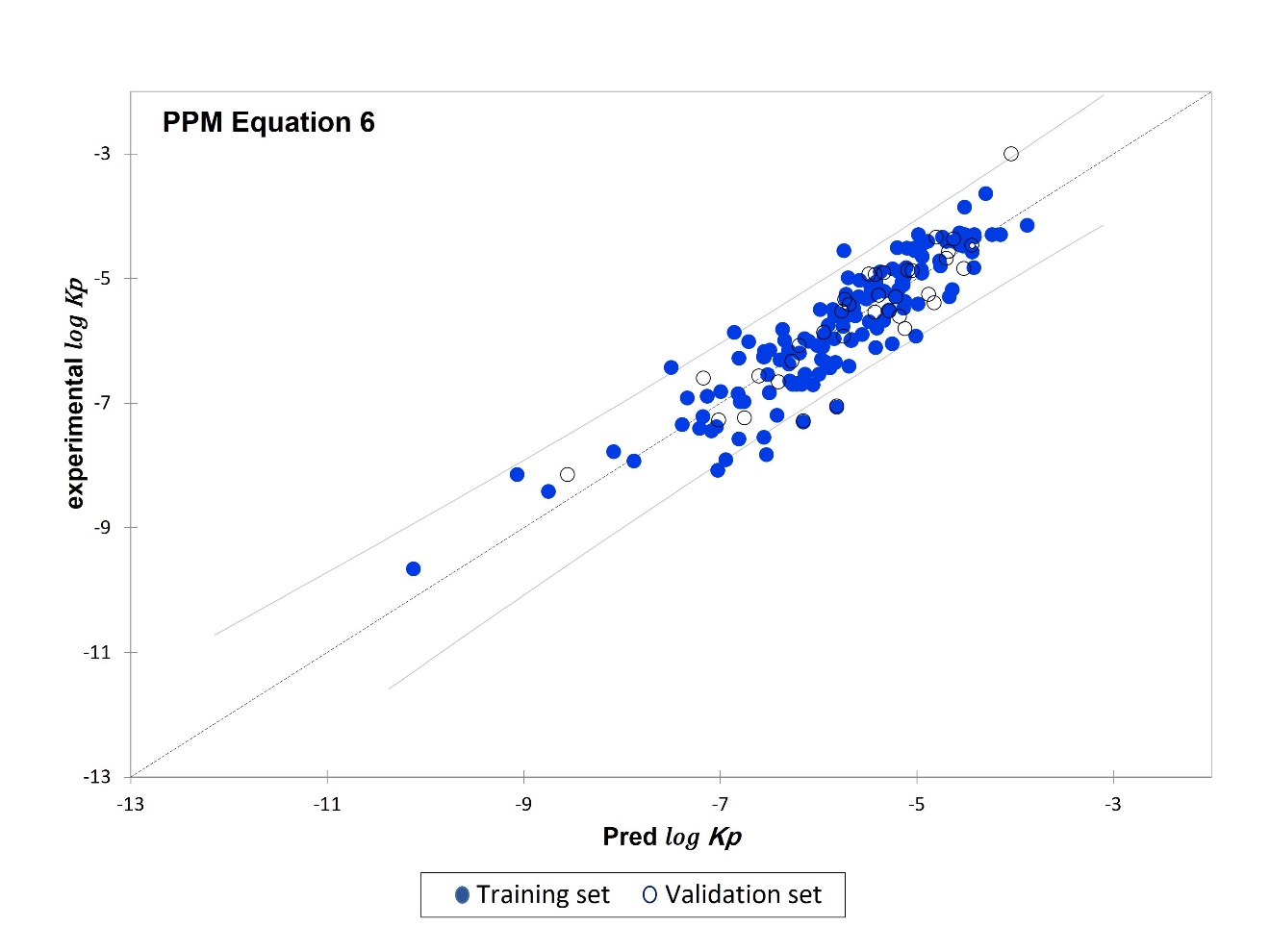


Figure S 4: Linear regression plot for Two - Parameter Partitioning Model (PPM) showing training set and validation set. Upper and lower dotted lines bound 95% confidence interval around the regression line (dotted middle line).

Table S 13: Cross-Validation of the PPM (equation 6) using four independent test.

| **Indicators** | **KNN(splitting of data, test vs train)** | | **LOOCV** | **K - fold CV** | **Repeated K - fold CV** | | **CV by Bootstrapping** | | |
| --- | --- | --- | --- | --- | --- | --- | --- | --- | --- |
|  | KNN(test) | KNN(train) |  |  | 3 times | 10times | N= 100 | N=500 | N=1000 |
| **R2** | 0.87 | 0.81 | 0.81 | 0.82 | 0.83 | 0.83 | 0.81 | 0.81 | 0.81 |
| **RMSE** | 0.39 | 0.48 | 0.48 | 0.46 | 0.47 | 0.47 | 0.48 | 0.48 | 0.47 |
| **MAE** | 0.31 | 0.38 | 0.38 | 0.38 | 0.38 | 0.38 | 0.38 | 0.38 | 0.37 |
| **PRE^*^** | -0.07 | -0.08 |  |  |  |  |  |  |  |

*Prediction Error Rate = RMSE/Mean

Section S 2: Training of Two - Parameter Partitioning Model (PPM) using EPI-Suite Estimated log $\boldsymbol{K}_{\boldsymbol{ow}}$ and log $\boldsymbol{K}_{\boldsymbol{aw}}$ and using Experimental log $\boldsymbol{K}_{\boldsymbol{ow}}$ and log $\boldsymbol{K}_{\boldsymbol{aw}}$

1. **PPM Trained on Experimental *log*** $\boldsymbol{K}_{\boldsymbol{ow}}$ **and *log*** $\boldsymbol{K}_{\boldsymbol{aw}}$

The PPM, based on a relationship of $\log K_{p}$ with a linear combination of *log* $K_{ow}$ and *log* $K_{aw}$, successfully described 85% of variation in the $\log K_{p}$ data.

$$\log K_{p}=-5.381 \left( \pm0.122 \right)+0.515 \left( \pm0.039 \right)\log K_{ow}+ 0.152 \left( \pm0.023 \right)\log K_{aw} (S2-1)$$

$$n=68, R^{2}=0.849, Adj. R^{2}=0.844, Q^{2}=0.834, RMSE=0.339$$

Here, the experimental values of $K_{ow}$ and $K_{aw}$ were used to train equation S2-1. Where, $n, R^{2}, Adj. R^{2}, Q^{2}, RMSE$ and $PRESS RMSE$ respectively denote number of experimental values of $\log K_{p}$, coefficient of determination, adjusted coefficient of determination, leave-one-out cross-validated $R^{2}$, root mean squared error and predicted residual error sum of squares, respectively.

1. **PPM Trained on EPI-Suite Estimated *log*** $\boldsymbol{K}_{\boldsymbol{ow}}$ **and *log*** $\boldsymbol{K}_{\boldsymbol{aw}}$

The PPM, based on a relationship of $\log K_{p}$ with a linear combination of *log* $K_{ow}$ and *log* $K_{aw}$, described 71% of variation in the $\log K_{p}$ data.

$$\log K_{p}=-5.212 \left( \pm0.122 \right)+0.387 \left( \pm0.03 \right)\log K_{ow}+ 0.163 \left( \pm0.012 \right)\log K_{aw} (S2-1)$$

$$n=175, R^{2}=0.712, Adj. R^{2}=0.708, Q^{2}=0.702, RMSE=0.589$$

Here, the values of $K_{ow}$ and $K_{aw}$, used to train equation S2-1, were estimated by the KOWWIN version 1.69 and HenryWin version 3.21 modules of US EPA EPI-Suite.

Table S 14: Selection of physicochemical variables for the estimation of skin permeability coefficients

| Variables Taken | Variables Retained | Highly influential variable | n | R^2^ | Adj.  R^2^ | Q^2^ | RMSE | PRESS RMSE | VIF | Internal validation |
| --- | --- | --- | --- | --- | --- | --- | --- | --- | --- | --- |
| log K_a-w_  log K_o-c_  log K_o-w_  log BCF  log D_w_  log D_eth_ | log K_a-w_  log K_o-w_ | log K_o-w_ | 175 | 0.844 | 0.84 | 0.83 | 0.439 | 0.462 | log K_o-w_ = 178.82  log K_a-w_  = 11.49  log K_o-c_ = 1106.14  log BCF  = 735.9  log D_w_  = 1026.18  log D_eth_  = 105.03 | R^2^ $\approx$ Q^2^  0.844 $\approx$ 0.826  RMSE $\approx$ PRESS RMSE  0.439 $\approx$ 0.462 |
| log K_o-w_  log D_w_ | log K_o-w_  log D_w_ | log K_o-w_ | 175 | 0.81 | 0.81 | 0.81 | 0.469 | 0.478 | log K_o-w_  =1.142  log D_w_ = 1.142 | R^2^ $\approx$ Q^2^  0.818 $\approx$ 0.810  RMSE $\approx$ PRESS RMSE  0.469 $\approx$ 0.478 |
| log K_o-w_,  log D_eth_ | log K_o-w_,  log D_eth_ | log K_o-w_ | 175 | 0.79 | 0.79 | 0.78 | 0.497 | 0.509 | log K_o-w_ ,  = 1.002  log D_eth_ = 1.002 | R^2^ $\approx$ Q^2^  0.795 $\approx$ 0.785  RMSE $\approx$ PRESS RMSE  0.497 $\approx$ 0.509 |

Table S 15: Training set for equation 8.

| S.N | Chemical | $\log K_{p}$  (Zhang Model) | $\log K_{p}$  (GC×GC Model) |
| --- | --- | --- | --- |
| 1 | decane | -2.60 | -2.77 |
| 2 | undecane | -2.35 | -2.54 |
| 3 | dodecane | -2.09 | -2.31 |
| 4 | methylcyclopentane | -3.84 | -3.76 |
| 5 | cyclooctane | -3.31 | -3.14 |
| 6 | cyclododecane | -2.27 | -2.31 |
| 7 | 3-methylcyclohexene | -3.95 | -3.74 |
| 8 | cyclonona-1,2-diene | -3.63 | -3.49 |
| 9 | fluoromethane | -5.27 | -5.40 |
| 10 | 1-fluorobutane | -4.66 | -4.62 |
| 11 | 1-fluoropentane | -4.43 | -4.30 |
| 12 | 1-fluorononane | -3.40 | -3.31 |
| 13 | tetrafluoromethane | -4.71 | -4.92 |
| 14 | 1-chlorooctane | -3.32 | -3.37 |
| 15 | 1,1,2-trichloroethane | -4.67 | -4.51 |
| 16 | hexachloroethane | -3.62 | -3.84 |
| 17 | γ-HCH | -4.28 | -3.50 |
| 18 | 1,3-butadiene, 1,1,2,3,4,4-hexachloro- | -3.37 | -3.33 |
| 19 | 1,3-cyclopentadiene, 1,2,3,4,5,5-hexachloro- | -3.25 | -3.15 |
| 20 | enflurane | -4.52 | -4.70 |
| 21 | 1-bromobutane | -4.29 | -4.12 |
| 22 | dibromomethane | -4.78 | -4.73 |
| 23 | tribromomethane | -4.41 | -4.36 |
| 24 | diiodomethane | -4.63 | -4.34 |
| 25 | 1,2-diiodethane | -4.03 | -4.24 |
| 26 | iodononane | -2.91 | -2.71 |
| 27 | benzene | -4.48 | -4.14 |
| 28 | benzene, propyl- | -3.86 | -3.79 |
| 29 | benzene, butyl- | -3.62 | -3.83 |
| 30 | benzene, pentyl- | -3.37 | -3.35 |
| 31 | benzene, octyl- | -2.59 | -2.62 |
| 32 | fluorobenzene | -4.53 | -4.53 |
| 33 | 1,3-difluorobenzene | -4.42 | -4.54 |
| 34 | 1,4-difluorobenzene | -4.43 | -4.57 |
| 35 | 1,3,5-trifluorobenzene | -4.29 | -4.53 |
| 36 | 1,2,3,5-tetrafluorobenzene | -4.23 | -4.56 |
| 37 | benzene, 1,3-dichloro- | -3.93 | -4.03 |
| 38 | benzene, 1,4-dichloro- | -3.99 | -4.09 |
| 39 | benzene, 1,2-dichloro- | -4.03 | -4.10 |
| 40 | benzene, hexachloro- | -3.11 | -3.06 |
| 41 | bromobenzene | -4.26 | -4.25 |
| 42 | 1,4-dibromobenzene | -3.87 | -3.89 |
| 43 | 1,3,5-tribromobenzene | -3.51 | -3.71 |
| 44 | iodobenzene | -4.20 | -4.21 |
| 45 | 1,3-diiodobenzene | -3.73 | -3.81 |
| 46 | 1,4-diiodobenzene | -3.71 | -3.97 |
| 47 | naphthalene | -4.28 | -4.06 |
| 48 | naphthalene, 1-methyl- | -3.97 | -3.75 |
| 49 | acenaphthylene | -4.07 | -3.99 |
| 50 | acenaphthene | -3.96 | -3.69 |
| 51 | dibenzofuran | -3.88 | -3.52 |
| 52 | fluorene | -3.80 | -3.51 |
| 53 | phenanthrene | -3.84 | -3.58 |
| 54 | pyrene | -3.83 | -3.75 |
| 55 | benz[a]anthracene | -3.47 | -3.09 |
| 56 | chrysene | -3.56 | -3.12 |
| 57 | PCB 28 | -3.15 | -3.33 |
| 58 | PCB 52 | -2.92 | -2.84 |
| 59 | PCB 101 | -2.77 | -3.31 |
| 60 | PCB 118 | -2.74 | -3.32 |
| 61 | PCB 138 | -2.61 | -2.88 |
| 62 | PCB 153 | -2.61 | -2.95 |
| 63 | PCB 180 | -2.46 | -2.80 |
| 64 | p,p'-DDE | -2.58 | -2.71 |

Table S 16: Validation set for equation 8.

| S.N | Chemical | $\log K_{p}$  (Zhang Model) | $\log K_{p}$  (GC×GC Model) |
| --- | --- | --- | --- |
| 1 | nonane | -2.85 | -3.00 |
| 2 | cyclohexadiene | -4.41 | -4.09 |
| 3 | 1,5,9-cyclododecatriene | -3.17 | -2.89 |
| 4 | sulfur hexafluoride | -4.46 | -4.59 |
| 5 | 1-chlorobutane | -4.36 | -4.28 |
| 6 | carbon tetrachloride | -4.17 | -4.24 |
| 7 | 1-bromooctane | -3.28 | -3.19 |
| 8 | hexabromoethane | -3.15 | -3.58 |
| 9 | 1-iodohexane | -3.67 | -3.47 |
| 10 | 1-iodobutane | -4.15 | -3.92 |
| 11 | toluene | -4.36 | -4.22 |
| 12 | benzene, decyl- | -2.08 | -2.15 |
| 13 | benzene, 1,2,4-trichloro- | -3.70 | -3.72 |
| 14 | 1,3-dibromobenzene | -3.88 | -3.92 |
| 15 | 1,2,3,5-tetrabromobenzene | -3.28 | -3.51 |

Table S 17: Cross-Validation of the GCxGC (equation 6) using four independent test.

| **Indicators** | **KNN(splitting of data, test vs train)** | | **LOOCV** | **K - fold CV** | **Repeated K - fold CV** | | **CV by Bootstrapping** | | |
| --- | --- | --- | --- | --- | --- | --- | --- | --- | --- |
|  | KNN(test) | KNN(train) |  |  | 3 times | 10times | N= 100 | N=500 | N=1000 |
| **R2** | 0.87 | 0.81 | 0.88 | 0.905 | 0.908 | 0.895 | 0.878 | 0.882 | 0.884 |
| **RMSE** | 0.39 | 0.48 | 0.24 | 0.23 | 0.24 | 0.24 | 0.26 | 0.25 | 0.25 |
| **MAE** | 0.31 | 0.38 | 0.19 | 0.19 | 0.195 | 0.195 | 0.20 | 0.20 | 0.19 |
| **PRE^*^** | -0.07 | -0.08 |  |  |  |  |  |  |  |


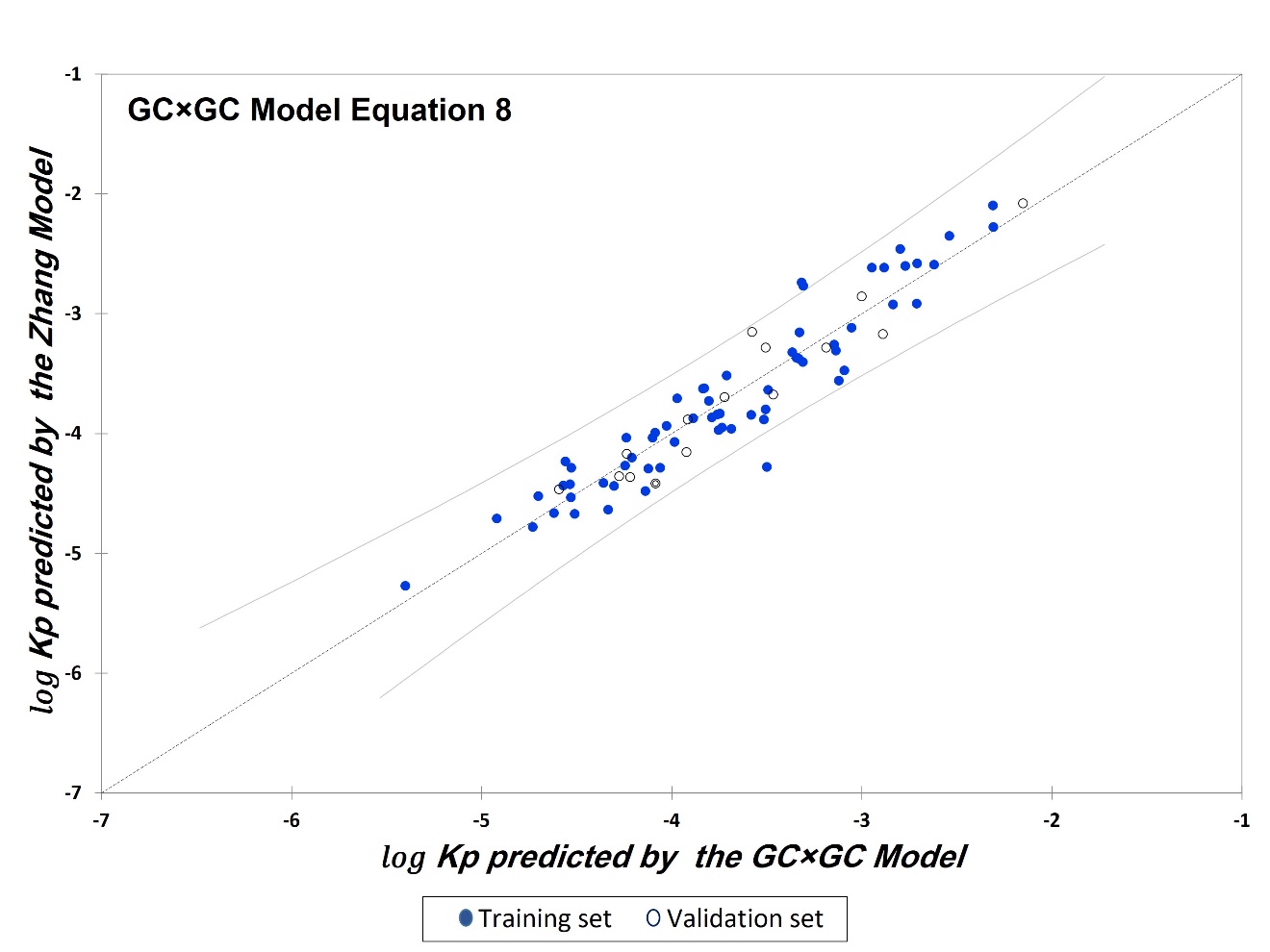


Figure S 5: Linear regression plot for the GC×GC Model showing training set and validation set. Upper and lower dotted lines bound 95% confidence interval around the regression line (dotted middle line).

Section S 3:**:** R Code used to perform statistical analysis on the skin data

#**Data Import and handling**

library(readxl)

Skin <- read_excel("Z:/RStudio_directory/Data/Skin.xlsx")

View(Skin)

class(Skin) #class of object Skin, returns data.frame

names(Skin) #retuns names of variables (columns)

dim(Skin) #returns number of rows and columns of data frame

str(Skin) #returns structure of variables in the data frame Skin

numericdataasm <- Skin[ ,2:8] #subset only numeric data

summary(Skin) #Summary statistics of variables in Skin

help(lm) #shows R Documentation for function lm()

# **regression model of DV logKp and IV L**.

m1 <- lm(logKp ~ L, data = Skin)

print(m1) #Prints coefficients of the model

summary(m1) #Prints summary of the model

r <- cor(Skin$logKp, Skin$L) #correlation coefficient of logKp and L

r ^ 2 #this is equal to r-squared in simple regression

ls(m1) #list of components in object class lm

m1$coefficients # returns coefficients of the model

m1$fitted.values[1:10] #a vector of fitted values

residuals <- m1$resid #a vector of residuals

coefficients(m1) # returns coefficients of the model

confint(m1) # returns a matrix of Confidence Interval for coefficients

plot(logKp ~ L, data = Skin) #scatter plot of logKp vs. L

abline(m1, col = "blue") #Add regression line to the scatter plot

anova(m1)

#**multiple regression model**

m2 <- lm(logKp ~ logKow + logKaw, data = Skin)

summary(m2) #summary of model m2

anova(m2) #anova table of model m2.

sum(anova(m2)$logKp) #sum of RSS and SSreg.

(175 - 1) * var(Skin$logKp) # To get the total sum of square of variable logKp we can multiply its’ variance by (n−1)

barplot(m2$coefficients) #bar plot of fitting coefficients

#**Standardized regression model**

m2.sd <- lm(scale(logKp) ~ scale(logKow) + scale(logKaw), data = Skin)

summary(m2.sd)

barplot(m2.sd$coefficients)#bar plot of standardized coefficients

# **part 2, Regression Diagnostics**

#install.packages("car")

#install.packages("alr3")

#install.packages("faraway")

#loading packages into working environment

library(car)

library(faraway)

library(alr3)

#scatter plot matrix from package car.

scatterplotMatrix(~ logKp + logKow + logKaw, data =Skin)

pairs(numericdataasm)

#**studentized residuals:**

er.std <- rstandard(m2)

#plot of studentized residuals.

plot(er.std, ylab="Standardized Residual", ylim=c(-3.5,3.5))

#adding horizental lines for cut of value of outliers.

abline(h =c(-2,0,2), lty = 2) #lty= line types

#determine which row is outlier(outside of cut of values of -2 and 2)

index <- which(er.std > 2 | er.std < -2)

#label Kp to points that are out of bounds

text(index-20, er.std[index] , labels = Skin$logKp)

#print row number of values that are out of bounds

index

#print Kp that are out of bounds

Skin$logKp[index]

#**Leverages:**

h <- influence(m2)$hat

#half normal plot of leverage from package faraway

halfnorm(h, ylab = "leverage")

# **Cook's distance:**

cutoff <- 4/((nrow(Skin)-length(m2$coefficients)-2))

#plot cook's distance

plot(m2, which = 4, cook.levels = cutoff)

#**influencePlot:** cook's distance, studentized residuals, and leverage in the same plot

influencePlot(m2, main="Influence Plot", sub="Circle size is proportial to Cook's Distance" )

FileName[c(write flagged Obs1, Obs2, Obs3, Obs4), c(1:3)] #retrerive the names of flagged observation

#4 diagnostic plots to intentify influential points

infIndexPlot(m2)

#residual vs. fitted value plot for Homoscedasticity (homogeneity of variance).

plot(m2$resid ~ m2$fitted.values)

#add horizental line from 0

abline(h = 0, lty = 2)

#residual vs. fitted value and all predictors plus test for curvature.

residualPlots(m2)

#residual plot vs. logKp

plot(m2$resid ~ Skin$logKp)

abline(h = 0, lty = 2)

#Normal Quantile to Quantile plot.

qqnorm(m2$resid)

qqline(m2$resid)

car::vif(m2) #variance inflation factor

**##Cross-Validation.**

#**1.The Validation set Approach:** load the following packages

**library**(tidyverse)

**library**(caret)

**library**(dplyr)

2. Split the data into training and test set. Load dplyr package before running this code as %>% might not work.

set.seed(123)

training.samples <- Skin$logKp %>%

createDataPartition(p = 0.8, list = FALSE)

train.data <- Skin[training.samples, ]

test.data <- Skin[-training.samples, ]

# Build the model

model <- lm(logKp ~ logKow + logKaw, data = train.data)

# Make predictions and compute the R2, RMSE and MAE

predictions <- model %>% predict(test.data)

data.frame( R2 = R2(predictions, test.data$logKp),

RMSE = RMSE(predictions, test.data$logKp),

MAE = MAE(predictions, test.data$logKp))

RMSE(predictions, test.data$logKp)/mean(test.data$logKp) #prediction error rate

View(training.samples)

View(train.data)

View(test.data)

write.csv(train.data, "train.csv")

write.csv(test.data, "test.csv")

write.csv(predictions, "pred.csv")

summary(model)

**##2. Leave one out cross validation – LOOCV**

# Define training control

train.control <- trainControl(method = "LOOCV")

# Train the model

model <- train(logKp ~ logKow + logKaw, data = Skin, method = "lm",

trControl = train.control)

# Summarize the results

print(model)

**##3. K-fold cross-validation**

# Define training control

set.seed(123)

train.control <- trainControl(method = "cv", number = 10)

# Train the model

model <- train(logKp ~ logKow + logKaw, data = Skin, method = "lm",

trControl = train.control)

# Summarize the results

print(model)

**##4.Repeated K-fold cross-validation**

# Define training control

set.seed(123)

train.control <- trainControl(method = "repeatedcv",

number = 10, repeats = 3)

# Train the model

model <- train(logKp ~ logKow + logKaw, data = Skin, method = "lm",

trControl = train.control)

# Summarize the results

print(model)

**##5. Bootstrap procedure**

# Define training control

train.control <- trainControl(method = "boot", number = 100)

# Train the model

model <- train(logKp ~ logKow + logKaw, data = Skin, method = "lm",

trControl = train.control)

# Summarize the results

print(model)

**##ESTIMATING STANDARD ERROR OF BETA USING BOOTSTRAPPING**

model_coef <- function(data, index){

coef(lm(logKp ~ logKow + logKaw, data = data, subset = index))

}

model_coef(Skin, 1:175)

library(boot)

boot(Skin, model_coef, 1000)

summary(lm(logKp ~ logKow + logKaw, data = Skin))$coef

**##PCA Analysis**

**#Load Libraries for visualization.**

library("FactoMineR")

library("factoextra")

**#Compute PCA**

res.pca <- prcomp(skinpca, scale = TRUE)

**#The output of the function PCA() is a list. To view it**

print(res.pca)

**#To get Eigenvalues / Variances**

eig.val <- get_eigenvalue(res.pca)

eig.val

**#Visualize eigenvalues (scree plot). Show the percentage of variances explained by each principal component.**

fviz_eig(res.pca)

fviz_eig(res.pca, addlabels = TRUE, ylim = c(0, 90))

**#Graph of variables**

var <- get_pca_var(res.pca)

var

**#Correlation circle**

fviz_pca_var(res.pca, col.var = "black")

**# Quality of representation**

library("corrplot")

corrplot(var$cos2, is.corr=FALSE)

**## Compute the correlation matrix**

cormat <- round(cor(Skin),2)

head(cormat)

write.csv(cormat, "cormat.csv")

## Create the correlation heatmap with ggplot2

library(reshape2) #The package **reshape** is required to **melt** the correlation matrix

melted_cormat <- melt(cormat)

head(melted_cormat)

# Get lower triangle of the correlation matrix

get_lower_tri<-function(cormat){

cormat[upper.tri(cormat)] <- NA

return(cormat)}

# Get upper triangle of the correlation matrix

get_upper_tri <- function(cormat){

cormat[lower.tri(cormat)]<- NA

return(cormat)}

upper_tri <- get_upper_tri(cormat) # Assign upper tri as an object

upper_tri # Print upper tri

##Reorder the correlation matrix

reorder_cormat <- function(cormat){

# Use correlation between variables as distance

dd <- as.dist((1-cormat)/2)

hc <- hclust(dd)

cormat <-cormat[hc$order, hc$order]}

# Reorder the correlation matrix

cormat <- reorder_cormat(cormat)

upper_tri <- get_upper_tri(cormat)

# Melt the correlation matrix

melted_cormat <- melt(upper_tri, na.rm = TRUE)

# Create a ggheatmap

ggheatmap <- ggplot(melted_cormat, aes(Var2, Var1, fill = value))+

geom_tile(color = "white")+

scale_fill_gradient2(low = "blue", high = "red", mid = "white",

midpoint = 0, limit = c(-1,1), space = "Lab",

name="Pearson\nCorrelation") +

theme_minimal()+ # minimal theme

theme(axis.text.x = element_text(angle = 45, vjust = 1,

size = 12, hjust = 1))+

coord_fixed()

# Print the heatmap

print(ggheatmap)

#Add correlation coefficients on the heatmap

#Use geom_text() to add the correlation coefficients on the graph

#Use a blank theme (remove axis labels, panel grids and background, and axis ticks)

#Use guides() to change the position of the legend title

ggheatmap +

geom_text(aes(Var2, Var1, label = value), color = "black", size = 4) +

theme(

axis.title.x = element_blank(),

axis.title.y = element_blank(),

panel.grid.major = element_blank(),

panel.border = element_blank(),

panel.background = element_blank(),

axis.ticks = element_blank(),

legend.justification = c(1, 0),

legend.position = c(0.6, 0.7),

legend.direction = "horizontal")+

guides(fill = guide_colorbar(barwidth = 7, barheight = 1,

title.position = "top", title.hjust = 0.5))
